# Longitudinal assessment of food insecurity status on the gut microbiome and metabolome of first-year college students

**DOI:** 10.1017/S0007114525103668

**Published:** 2025-06-17

**Authors:** Alex E. Mohr, Paniz Jasbi, Irene van Woerden, Devin A. Bowes, Jinhua Chi, Haiwei Gu, Meg Bruening, Corrie M. Whisner

**Affiliations:** 1 College of Health Solutions, Arizona State University, Phoenix, AZ, USA; 2 Center for Health through Microbiomes, Biodesign Institute, Arizona State University, Tempe, AZ, USA; 3 Systems Precision Engineering and Advanced Research (SPEAR), Theriome Inc., Phoenix, AZ, USA; 4 Department of Community and Public Health, Idaho State University, Pocatello, ID, USA; 5 Department of Environmental Health Sciences, Arnold School of Public Health, University of South Carolina, 921 Assembly Street, Columbia, SC 29208, USA; 6 Center for Translational Science, Florida International University, Port St. Lucie, FL, USA; 7 Department of Nutritional Sciences, College of Health and Human Development, Pennsylvania State University, University Park, PA, USA

**Keywords:** Food security, Adolescence, Gut microbiota, Metabolome, Multi-omics, Systems biology, Stability

## Abstract

Food insecurity affects the health of college-aged individuals, but its impact on the gut microbiome (GM) over time is poorly understood. This study explored the association between food insecurity and the GM in eighty-five college students, identifying microbial taxa, metabolites and pathways linked to food security status and examining GM stability and microbe–metabolite interactions. Longitudinal GM and metabolomic data were collected from first-year students over an academic year, encompassing periods of variable food security status. Participants were categorised into three groups: food insecure (FI, *n* 13), food secure (FS, *n* 44) and variable (VAR, *n* 28) status. GM composition varied significantly between FS classifications (Bray–Curtis dissimilarity, *P* ≤ 0·005). Stability analysis revealed correlations between stability scores and microbial features, pathways and metabolites. Specific microbes (e.g. *Bifidobacterium* species, *Faecalibacterium prausnitizii D* and *Lachnospiraceae*), pathways (energy and microbial turnover) and metabolites (cadaverine, N-acetylcadaverine, putrescine, testosterone sulfate and creatine) associated with FI status were identified. Multi-omic integration revealed metabolic pathways influenced by differentially abundant microbial species and co-occurring fecal metabolites in FI participants related to the microbial production of polyamines, detoxification and energy metabolism. The transition from FS to FI showed no significant differences at specific taxonomic, functional or metabolite levels. This study uncovers complex interactions between food security, GM composition and metabolism. Significant differences were found in microbial community variability and metabolic pathways associated with food security status, but the transition from food security to insecurity disrupted the GM without clear taxonomic or functional distinctions, emphasising the need for further research into these mechanisms.

Food insecurity is an increasing public health problem that disproportionally affects vulnerable populations across the lifespan. Despite targeted efforts, food insecurity continues to be prevalent in the USA, occurring in 12·8 % of households as of 2021^(1)^. Defined as a socio-cultural construct tied to the lack of consistent access to healthy food^(2)^, food insecurity is particularly problematic amongst college-aged individuals with prevalence levels several times higher than the national average^(3)^. Food insecurity in this population is correlated with poor physical and mental health, dietary and academic outcomes^(3-7)^. During the formative period of emerging adulthood (the general age of college students), food insecurity has the potential to significantly disrupt an individual’s health trajectory, leading to suboptimal health that may persist into later life^(8)^.

Food insecurity shares multiple characteristics with dietary behaviours involving nutrient or food deprivation, such as energy restriction, intermittent fasting and disordered eating^(9-11)^. However, it is important to recognise that the psychological mechanisms surrounding food insecurity, including anxiety and depression related to food access, are distinct from voluntary dietary practices (e.g. energy restriction)^(12)^. Moreover, a direct comparison of the aforementioned dietary behaviours with food insecurity for factors such as energy intake, nutrient profile and other related health considerations may not be appropriate. Therefore, more research in this area is greatly warranted, particularly longitudinal assessments in tandem with systems biology approaches to unravel the complex impact of food insecurity on individual health. In this context, the gut microbiome (GM), comprising the diverse community of microbes inhabiting the gastrointestinal tract, has emerged as a valuable and elucidating factor, responsive to dietary pressures akin to food security status. For example, evidence supports that energy restriction, intermittent fasting and disordered eating influence the ecological landscape of the GM, modulating microbial competitiveness favouring particular taxa and metabolic pathways^(13-16)^. The metabolites synthesised or modified within this environment have also been reported to play a crucial role in corresponding health implications for the host^(17)^. For instance, microbial-generated metabolites have been suggested as a mediator between an altered GM and behaviour associated with anorexia nervosa^(15)^. Consequently, the GM and metabolome represent essential complementary omic layers, poised to offer predictive biomarkers and co-occurrent signatures for capturing pathological variation over time.

Previously, we established distinct microbial and metabolic signatures in food-insecure (FI) individuals compared with food-secure (FS) counterparts in a college-based population^(18)^. Our findings suggested that compared with FS students, the GM of FI students exhibited greater within-sample differences and estimated metabolic pathway activity, particularly related to hydrolysis reactions, energy substrate biosynthesis and macronutrient metabolism. Additionally, we observed elevated metabolites (i.e. picolinic acid, phosphocreatine, 2-pyrrolidinone) associated with energy transfer and gut–brain axis communication with food insecurity^(18)^. While this work was cross-sectional, these results indicate that the architecture of an FI GM may be primed to handle periods of deprivation and encode for the production/trans-formation of metabolic products relevant to gut–brain communication. However, the ecological stability of microbial communities under dietary pressure remains unclear, as within-subject longitudinal variation depends on factors like host lifestyle or sudden changes in nutritional status^(19)^. Furthermore, it is important to understand how functional attributes and metabolic end-products of the GM may shift over time depending on variable or fixed food security status.

As a continuation of our research to uncover the influence of food insecurity status on the GM and its functional output^(18)^, we assembled longitudinal GM and metabolomic data from first-year college students, capturing a timeframe that accurately aligns with the academic year and is known to induce variability in food security status^(3)^. We categorised participants into three groups: those with a fixed status of either FI or FS and those with a variable (VAR) status throughout the study period. Our objectives were to identify specific taxa, metabolites and functional pathways in the gastrointestinal tract associated with food security status, while also shedding light on GM stability and meaningful microbe–metabolite co-occurrence signatures that may influence host health. This investigation represents an important, early step in deepening our understanding of the underlying mechanisms linking food security status to GM dynamics and metabolic profiles, which may ultimately provide valuable insight to improve health outcomes in this vulnerable population.

## Methods

### Participants and study design

The devilWASTE study was a sub-study of the Social impact of Physical Activity and nutRition in College (SPARC) study, which sought to analyse relationships between lifestyle factors, weight outcomes and the social networks of first-year college students^(20)^. DevilWASTE participants were recruited from the SPARC cohort from six dorms across three different Arizona State University campuses. Participants in devilSPARC were recruited from one dorm on each of the three campuses. Given participants lived on campus, all participants were required to have a meal plan. All participants had prepaid for at least 8 meals per week. The exclusion criteria for devilWASTE included age less than 18 years, certain gastrointestinal conditions such as malabsorptive diseases, history of eating disorders, antibiotic use 3 months prior to study visits and conditions that affect the microbiome including HIV infection, diabetes and high blood pressure. Inclusion criteria included living in a residence hall at Arizona State University, English speaking and participation in the SPARC study. Eligible participants provided written informed consent before enrollment. The devilWASTE study and the parent SPARC study were approved by the Arizona State University Institutional Review Board.

### Data collection

Recruitment for devilWASTE took place during the academic year starting in August 2015 and data collection continued through May 2016. Stool samples were collected at up to three of the four SPARC study time points (beginning and end of fall and spring semesters). In the present analysis, only individuals who provided a stool sample at the start of the Fall semester (T1), either at the end of the Fall or start of the Spring semester (T2) and at the end of the Spring semester (T3) were retained to support this longitudinal analysis. At each time point, anthropometrics, physical activity and diet information were collected. At baseline socio-demographic characteristics including age, sex, race and ethnicity were collected. Participant Pell grant status for the Fall 2015 and Spring 2016 semesters was acquired from university records.

Anthropometrics were obtained by trained research staff using Seca 869 scales (Seca, USA) for weight (kg), Seca 217 stadiometers (Seca, USA) for height (cm) and flexible, tension spring-loaded Gulick measuring tapes for waist (cm) and hip (cm) circumference. These measurements were taken at each of the four devilSPARC study visits and completed up to three times at each visit to ensure accuracy. The two measures that were within 0·5 kg and 0·5 cm for weight and height, respectively, were averaged for final measurements. If none of the first three measurements were within 0·5 units of each other, another set of up to three measurements were taken. Similarly, the two waist and hip measures within 0·5 cm were averaged for final measures, and if none were within 0·5 cm another set of up to three measurements was obtained. Participant BMI were calculated and reported in kg/m^2^ while waist-to-hip ratios were calculated as waist divided by hip.

Questionnaires were used to evaluate lifestyle behaviours. Physical activity was assessed with the Godin–Shephard Leisure-Time Physical Activity Questionnaire which categorises and quantifies activity into vigorous, moderate and light physical activity^(21)^. Sedentary behaviour was assessed with an additional question: ‘Yesterday, how much time did you spend in front of a screen (excluding time in class and being physically active)?’^(22)^. Participants selected a response from a range of zero to six hours. Self-reported dietary intake was reported using the National Cancer Institute Dietary Screener Questionnaire which assesses the consumption frequency of key food items and food groups^(23)^. This tool does not estimate energy intake but rather tracks consumption of food group categories such as fruits and vegetables, high-fat and processed foods, added sugar, dietary fibre and whole grain intake. Weekly alcohol intake was assessed by the number of drinks consumed weekly by asking: ‘For each day of the week in the calendar below, indicate the number of alcoholic drinks typically consumed on that day (Only if yes to alcohol is selected).’ The dropdown ranged from 1 to 15 drinks for all days of the week. For capturing depression^(24)^ and perceived stress^(25)^, students completed previously validated questionnaires with robust test–rest correlations (0·89 and 0·74, respectively) in a similar college-aged population^(6)^.

Food insecurity was measured using the US Department of Agriculture 6-item food insecurity screener^(26)^. The time frame in the validated question was adapted and the framing of the question changed from ‘we’ to ‘I’, as has been done by others^(27)^. Of the six items administered, participants who provided an affirmative answer to two or more questions were categorised as FI in the past month. Participants were then distributed into three groups: those with a fixed status of FI or FS and those with a VAR status throughout the study period.

### Fecal sample collection and DNA extraction

Research staff delivered faecal sample collection kits to the residence halls of eligible participants. As previously noted, faecal samples were collected at up to three timepoints for each participant. Participants were asked to report any medication and supplement use within the last 3 months; if participants had taken any antibiotics, antifungals or probiotics within the previous 3 months, a faecal sample was not obtained. Research staff picked up the faecal samples within 30 min of a participant’s reported bowel movement and transported them to the laboratory where they were frozen at −80°C until further processing. Frozen samples were thawed at 4°C, and wet weight was recorded to the nearest 0·01 g after subtracting the weight of faecal collection materials. DNA was extracted from approximately 300 mg of faeces and collected from the centre of the sample, using a modified version of the manufacturer protocol (MoBio Power Soil DNA Isolation Kit #12888-100, MoBio). Per manufacturer recommendations, a heating step of 65°C for 10 min was added to the protocol to reduce the influence of inhibitors commonly found in faeces and increase DNA yield. DNA concentration and quality were quantified using the QIAxpert System (Qiagen) according to manufacturer instructions.

### Fecal microbiome sequencing

High-throughput genomic sequencing of the 16S rRNA gene was performed at the Biodesign Institute at Arizona State University in Tempe, Arizona using Illumina miSeq technology after ligating 515F and 806R primers and Illumina adapters via polymerase chain reaction. Negative controls were included and run with the study samples. A detailed report of methods to prepare and sequence DNA has been published^(18,28)^.

Due to the complex nature of the devilWASTE study design, a large number of participants and low-quality sequencing for a few samples in initial runs, three sequencing runs were conducted. In cases where samples were sequenced multiple times, files were merged after performing quality control. This method has been supported by the expert census from the Quantitative Insights Into Microbial Ecology 2 (QIIME2) software development team^(29)^, the bioinformatic pipeline used in the present analysis. Overall, the 16S rRNA sequencing produced a total of 22 628 375 reads across the three runs with a median of 46 238 (run 1), 33 395 (run 2), and 77 808 (run 3) reads per sample. Paired-end, demultiplexed data were imported and analysed using QIIME 2 software version 2021.8. Upon examination of sequence quality plots, base pairs were trimmed at position 20 and truncated at position 240 and were run through DADA2 (each run separately to prevent error modelling) to remove low-quality regions and construct a feature table using amplicon sequence variants (ASV). Next feature tables and representative sequences from the three runs were merged using the functions ‘feature-table merge’ and ‘feature-table merge-seqs’, respectively. Parameters were set to ‘sum’ for ‘–p-overlap-method’ to pool all features from duplicated samples in one and summarise all identical features in that sample. Taxonomy was assigned by first constructing a classifier against the Genome Taxonomy Database (r.202)^(30)^ using a naive Bayes approach via the q2-feature-classifier. Refined taxonomic classification was produced by assembling bespoke taxonomic weights with q2-clawback to achieve benchmarked species-level resolution^(31)^. A phylogenic tree was then constructed using the fragment insertion plugin. A rarefaction threshold was assessed at 8000 and used to impute high-quality reads and normalise for uneven sequencing depth between samples^(32)^. A *phyloseq* (v1.38.0) object was created, and downstream analyses and visualisations were performed in R (v.4.1.2). Sequences were removed including mitochondrial and plant DNA. In addition, any remaining singleton ASV across samples were removed. Alpha-diversity was calculated using observed ASV and phylogenetic diversity metrics. Beta-diversity was assessed using the Bray–Curtis dissimilarity metric. Estimated functional potential of the overall bacterial community was surveyed via the Phylogenetic Investigation of Communities by Reconstruction of Unobserved States 2 (PICRUSt2) algorithm (v2.4.2)^(33)^. Pathway abundances were inferred based on structured pathway mappings of Enzyme Commission gene families to the MetaCyc database^(34)^.

### Fecal metabolomics

Acetonitrile, methanol (MeOH), ammonium acetate and acetic acid, all liquid chromatography-MS grade were purchased from Fisher Scientific. Ammonium hydroxide was bought from Sigma-Aldrich. DI water was provided in-house by a Water Purification System from EMD Millipore. PBS was bought from GE Healthcare Life Sciences. The standard metabolite compounds were purchased from Sigma-Aldrich and Fisher Scientific.

Briefly, each faecal sample (~20 mg) was homogenised in 200 μl MeOH:PBS (4:1, v:v, containing 1810·5 μM ^13^C_3_-lactate and 142 μM ^13^C_5_-glutamic Acid) in an Eppendorf tube using a Bullet Blender homogeniser (Next Advance). Then, 800 μl MeOH:PBS (4:1, v:v, containing 1810·5 μM ^13^C_3_-lactate and 142 μM ^13^C_5_-glutamic Acid) was added, and after vortexing for 10 s, the samples were stored at −20°C for 30 min. The samples were then sonicated in an ice bath for 30 min. The samples were centrifuged at 14 000 RPM for 10 min (4°C), and 800 μl supernatant was transferred to a new Eppendorf tube. The samples were then dried under vacuum using a CentriVap Concentrator (Labconco). Prior to MS analysis, the obtained residue was reconstituted in 150 μl 40 % PBS/60 % acetonitrile. A quality control sample was pooled from all the study samples.

The untargeted liquid chromatography-MS metabolomics method used here was modelled after that developed and used in a growing number of studies^(28,35)^. Briefly, all liquid chromatography-MS experiments were performed on a Thermo Vanquish UPLC-Exploris 240 Orbitrap MS instrument. Each sample was injected twice, 10 μl for analysis using negative ionisation mode and 4 μl for analysis using positive ionisation mode. Both chromatographic separations were performed in hydrophilic interaction chromatography mode on a Waters XBridge BEH Amide column (150 × 2·1 mm, 2·5 μm particle size, Waters Corporation). The flow rate was 0·3 ml/min, auto-sampler temperature was kept at 4°C and the column compartment was set at 40°C. The mobile phase was composed of Solvents A (10 mM ammonium acetate, 10 mM ammonium hydroxide in 95 % H_2_O/5 % acetonitrile) and B (10 mM ammonium acetate, 10 mM ammonium hydroxide in 95 % acetonitrile/5 % H_2_O). After the initial 1 min isocratic elution of 90 % B, the percentage of Solvent B decreased to 40 % at t = 11 min. The composition of Solvent B was maintained at 40 % for 4 min (t = 15 min), and then the percentage of B gradually went back to 90 %, to prepare for the next injection. Using mass spectrometer equipped with an electrospray ionisation source, we will collect untargeted data from 70 to 1050 m/z.

To identify peaks from the MS spectra, we made extensive use of the in-house chemical standards (~600 aqueous metabolites), and in addition, we searched the resulting MS spectra against the Human Metabolome Database library, Lipidmap database, METLIN database, as well as commercial databases including mzCloud, Metabolika and ChemSpider. The absolute intensity threshold for the MS data extraction was 1000, and the mass accuracy limit was set to 5 ppm. Identifications and annotations used available data for retention time, exact mass (MS), MS/MS fragmentation pattern and isotopic pattern. We used the Thermo Compound Discoverer 3·3 software for aqueous metabolomics data processing. The untargeted data were processed by the software for peak picking, alignment and normalisation. To improve rigour, only the signals/peaks with CV < 20 % across QC pools and the signals showing up in > 80 % of all the samples were included for further analysis. Chemical taxonomy was obtained by querying metabolome features against the most recent version of the Human Metabolome Database (v.5·0)^(36)^. A Canberra distance matrix was computed on the metabolomics feature table, as previously described^(37)^.

### Statistical analysis

Anthropometric, behavioural and dietary data from participant’s was first assessed for normality using QQ plots and Shapiro–Wilk’s test. Differences in baseline values between the three FS statuses were then analysed by ANOVA or Kruskal–Wallis tests, depending on normality. Potential differences in anthropometric, behavioural and dietary data at each of the three time points between the three FS classifications were assessed by ANOVA or Kruskal–Wallis tests with post hoc comparisons, where appropriate.

To ensure that the study had sufficient power to detect differences in GM composition across the food security groups, we conducted a power analysis using the Xmcupo test from the *HMP* package (v2.0.2.). The Xmcupo test applies a Dirichlet-Multinomial model, which is particularly suited for over-dispersed microbiome data. The Xmcupo test demonstrated sufficient statistical power to detect significant differences in microbial community composition between the groups. The analysis returned an Xmcupo statistic of 4207·375 (*P* < 0·001), indicating a highly significant difference in microbiome composition across food security statuses. This result confirms that, despite the unequal group sizes, the study design provides adequate power to detect meaningful differences in gut microbial diversity and composition between the food security groups.

For analysis of the GM, features and estimated functional pathways that were not present in at least 10 % of samples were removed. First, a permutation test for homogeneity in multivariate dispersion (PERMDISP) was conducted using the ‘betadisper’ function in the *vegan* package (v2.6.2.) to compare dispersion across FS classification. Next, calculated intra-individual differences for the microbiome, estimated functional pathways and faecal metabolome and *α* diversity metrics were tested for the effect of time, group and their interaction using linear-mixed effect modelling using the *nLME* package (v3.1.153.). To assess feature stability, we computed Bray–Curtis dissimilarity (for species and estimated pathways) and Canberra distance (for the fecal metabolome) using the *ecodist* package (v2.0.9.) for each participant across the three sample collection time points. We then calculated a stability score for each feature as 1 minus its Bray–Curtis (or) Canberra value (i.e. ‘1 – dissimilarity’). Because a smaller dissimilarity value indicates higher similarity across time points, this approach yields a stability measure that ranges from 0 (least stable) to 1 (most stable). This metric has been previously described^(19,38)^ but here serves to highlight how consistently a given feature’s abundance remains within an individual over multiple time points. We then pooled all feature-level observations (i.e. each species, pathway or metabolite in each participant) to perform a single Spearman’s rank correlation (*ρ*) comparing the distribution of stability scores to the distribution of log-transformed abundances within each data type (microbial species, pathways or metabolites). Next, to determine whether the slope (relationship) between stability and abundance differed by FS status, we fitted separate linear regression models for each data type (estimates reflect the average across all features within that data type). In these models, individual feature observations were nested within each FS classification, and FS status was treated as an interaction term with the stability measure. Where a significant interaction was observed, we conducted post hoc pairwise comparisons between FS categories to test for differences in the slope of stability *v*. abundance. If significant interactions were detected, pairwise contrasts were used to detect significant differences in beta coefficients between the FS classifications. Permutational multivariate ANOVA (PERMANOVA) models were constructed for Bray–Curtis dissimilarity metrics (microbiome and estimated functional pathways) in the *vegan* package testing the effects of the individual (nested factor), FS status, time and the interaction between these factors, controlling for sex and baseline BMI (permutation *n* 999). Differential abundance analyses for microbial features at the phylum, family and species level and estimated functional pathways were performed using the *MaAsLin2* package with default parameters (v1.12.0)^(39)^. To detect differences in features between the three FS statuses, we built linear mixed models with FS as the reference group that included time, the covariates of sex and baseline BMI and the participant as a random factor.

To assess differences in metabolite abundances between the three FS statuses, faecal metabolome samples from all time points were pooled, and a covariate-adjusted general linear model, accounting for time, sex and baseline BMI, was assembled with FS status used as the reference group. Significant features were then selected and used to construct a supervised partial least squares-discriminant analysis (PLS-DA) as a model predictive of FI and FS status. Pathway and enzyme topology and enrichment models were analysed between groups using MetaboAnalyst software^(40)^. For pathway analysis, impact was calculated using a hypergeometric test, while significance was determined using a test of relative betweenness centrality. All metabolomics data were log10-transformed, and Pareto scaled to approximate normality prior to analysis.

For multi-omic analysis, we integrated significant microbial taxa from the linear mixed models and the reliably detected and annotated faecal metabolomic data with graph-guided fused least absolute shrinkage and selection operator (GFLASSO) regression (R package: *GFLASSO*, v0.0.0.9000). Using this correlation-based network approach, significant microbial features were entered as the predictor variable and faecal metabolites as the response variable. Solution parsimony was determined by an unweighted (i.e. presence or absence of association by imposing a correlation threshold) network structure. For our purposes, we imposed a Spearman’s rank correlation of *ρ* = 0·80 to provision for balance between running time and network density. The regularisation and fusion parameters were determined from the smallest root mean squared error estimate via cross-validation, accounting for interdependencies among microbial features. The tested parameters encompassed all combinations between λ and γ with values ranging from 0 to 1 (inclusive) in step increments of 0·1. GFLASSO coefficient matrices were constructed using a threshold coefficient of > 0·02 to discern the strongest associative signals. The highest beta coefficients detected from GFLASSO models were further assessed by performing Spearman correlations of select microbial features with the response variables.

Where appropriate, statistical models accounted for baseline BMI and sex as covariates with participants as a random effect. A *P*-value of 0·05 was used to denote statistical significance and *P*-value adjustments (*Q*) were performed where appropriate using the Benjamini–Hochberg procedure. A *Q*-value < 0·10 was considered statistically significant. All statistical analyses were performed in the R environment (v4.1.2.).

## Results

### Participant characteristics

**Figure 1.**
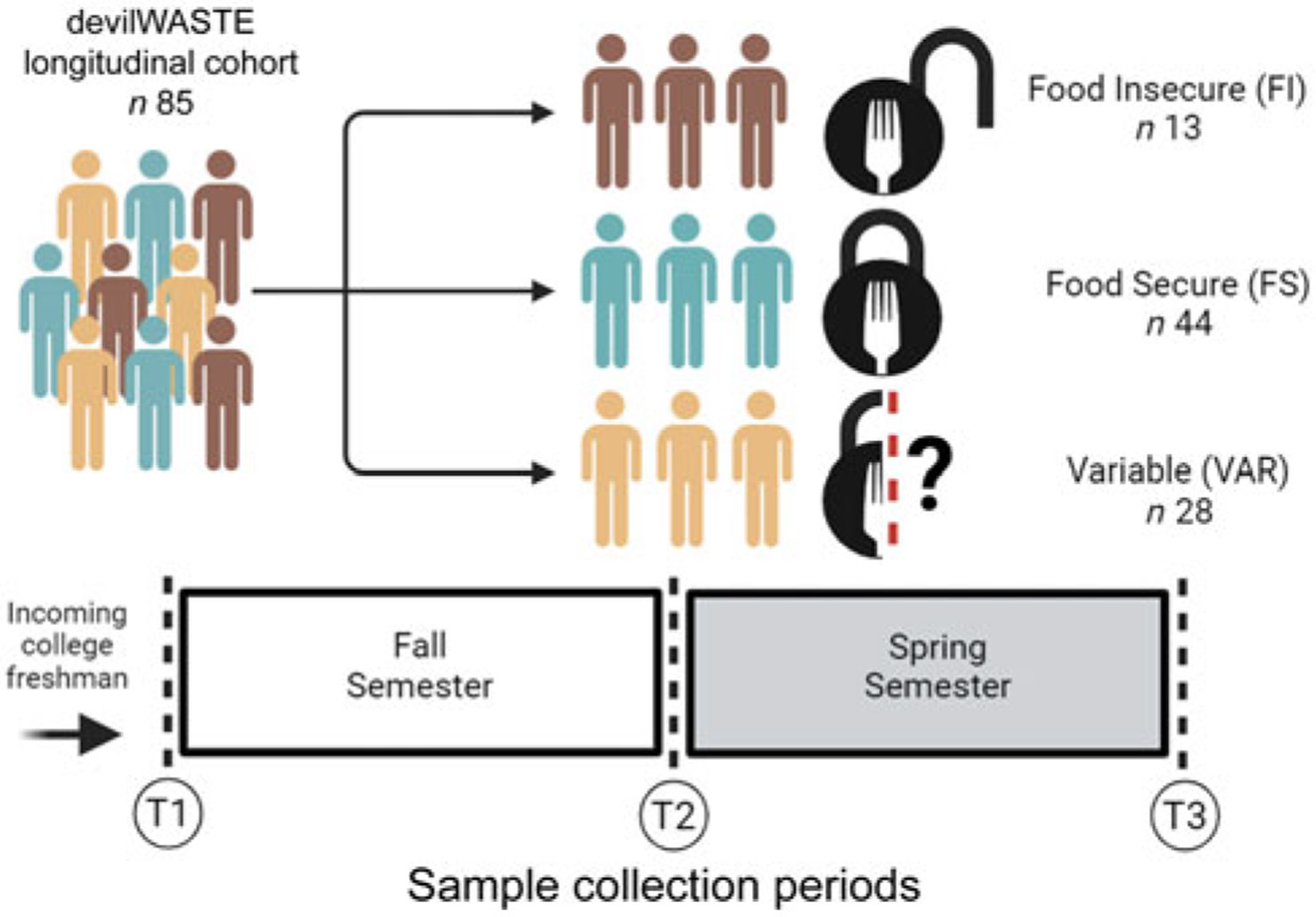
Overview of the study design and sample collection periods used in the present analysis.

In total, eighty-five participants from the previously described parent study^(20)^ provided a full stool sample at the requisite time points and were included in this analysis (Figure 1). No significant differences between FS classification at baseline (beginning of the Fall semester) were detected for age, body weight, waist circumference, waist/hip ratio or BMI (ANOVA/Kruskal–Wallis tests, *P* ≥ 0·085); nor for self-reported lifestyle behaviours, including moderate to vigorous physical activity, screen time, stress, sleep and dietary factors (ANOVA/Kruskal–Wallis tests, *P* ≥ 0·079; Table 1). There was a significant difference in depression and total alcohol consumption (Kruskal–Wallis tests, *P* ≤ 0·011), with FI individuals displaying greater initial levels compared to FS individuals (pairwise comparison, *P* ≤ 0·05). As a proxy for economic status, we did not detect a significant difference between classifications for Pell grant status (Fisher’s exact test, *P* = 0·981). No significant time or interaction effects were detected for any anthropometric or behavioural variable (*P* ≥ 0·085; online Supplementary Table S1).

### Community structure and stability of gut microbes, function and metabolome display variability by food security status

Covariate controlled and nested adonis analysis revealed significant individual variance over time between the three food security classifications (PERMANOVA, *R*^2^ = 0·194, *P* = 0·001), with the individual effects of group (*R*^2^ = 0·017, *P* = 0·001) and time (*R*^2^ = 0·005, *P* = 0·001) explaining less overall variance. This was not the case for estimated functional pathways, which was not significant for any factor as above (*P* = 0·180) or the individual variance over time for the faecal metabolome (*P* = 0·304). However, the faecal metabolome was significant for the individual effects of group (*R*^2^ = 0·015, *P* = 0·001) and time (*R*^2^ = 0·005, *P* = 0·001).

**Figure 2.**
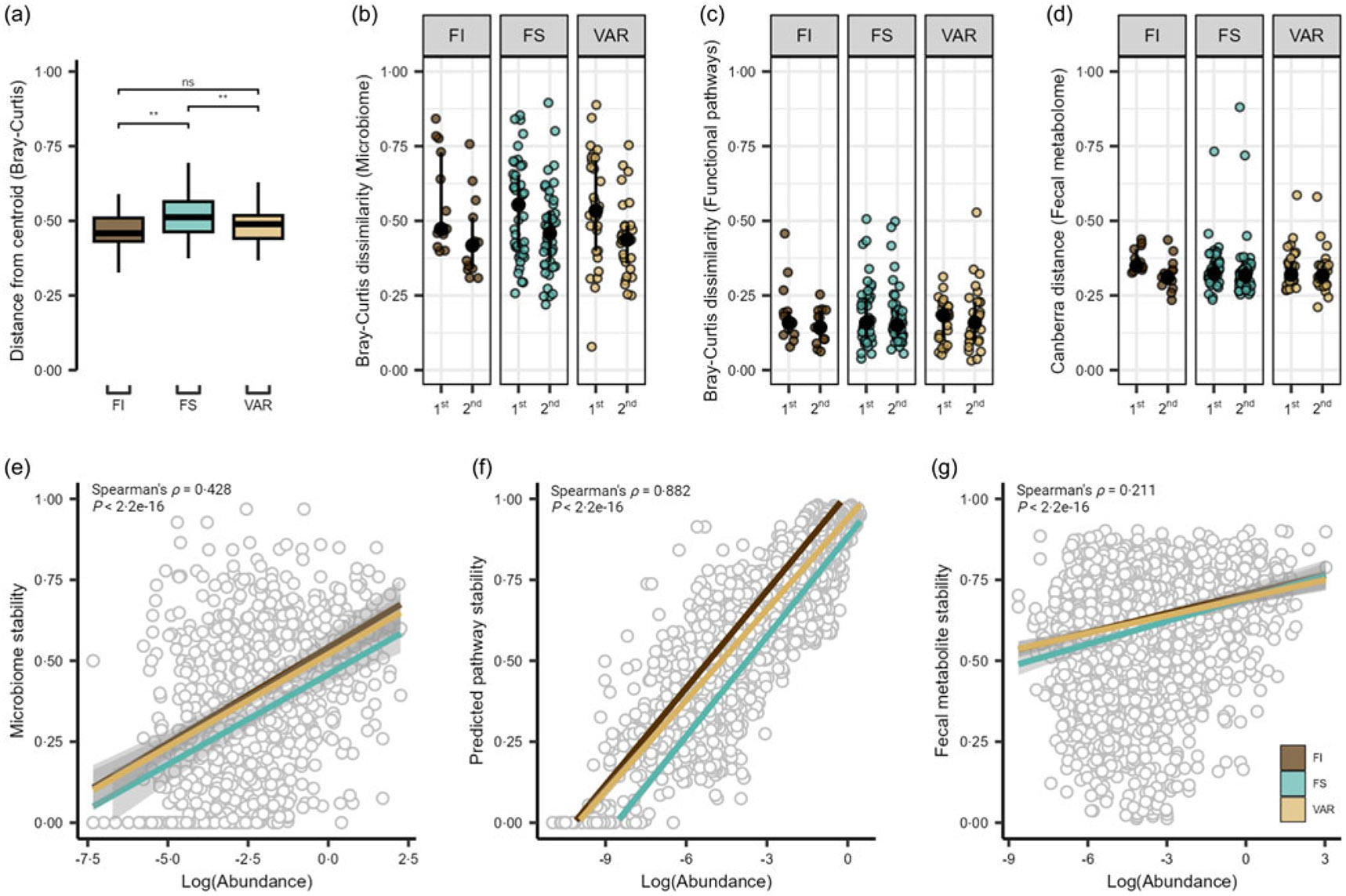
Gut microbiome and metabolome of college students by food security status. (a) Beta dispersion of Bray–Curtis dissimilarity for food insecure (FI), food secure (FS) and variable food security (VAR) classifications. NS = non-significant, ** *P* ≤ 0·005. Intra-individual shifts over the duration of the study period showing the 1st (time point 1 *v* 2) and 2nd (time point 1 *v* 3) time comparisons for the (b) microbiome, (c) estimated functional pathways and (d) faecal metabolome. Scatter plots show Spearman’s rank correlation coefficients and linear regression slopes by FS status for (e) gut microbiome, (f) estimated functional pathway and (g) faecal metabolome stability of each feature (1·0 = most stable, 0 = least stable) *v*. log-transformed abundance.

There were no significant time or interaction effects for the surveyed α diversity metrics, observed ASV and phylogenic diversity (*P* ≥ 0·122; online Supplementary Figure S1A and B). Comparison of distributions of Bray–Curtis dissimilarity with a total of 3570 ASV across 255 samples revealed significant within-group differences by food security status (ANOVA, *P* = 0·004), with the GM of FS participants showing greater overall variability compared with FI and VAR participants (pairwise comparison, *P* ≤ 0·008; Figure 2(a)). Assessing Bray–Curtis intra-individual dissimilarity, time had a significant effect (*P* = 1·0e-04), with the overall GM community appearing to display greater dissimilarity between the first two timepoints and then becoming more similar to baseline by the third time point (Figure 2(b)). In contrast, the pooled 334 estimated microbial functional pathways were not significant by any factor using Bray–Curtis dissimilarity (linear-mixed effect, *P* ≥ 0·468; Figure 2(c)), suggestive of the established conserved functional pathways compared to taxonomic membership in the GM^(41)^. For the faecal metabolome, the reliably detected 554 metabolites (i.e. QC CV < 20 % and relative abundance > 1000 in 80 % of samples) displayed no significant effects (*P* ≥ 0·173; Figure 2(d)) via assessment of the Canberra distance.

We next extended these results to explore the stability of individual microbial features and estimated functional pathways and faecal metabolites. Pooling feature-level values across individuals within each data type, we noted moderate, large and small correlations between stability scores and log(abundance) for microbial species (Spearman’s *ρ* = 0·422, *P* = 2·2e-16; Figure 2(e)), estimated pathways (Spearman’s *ρ* = 0·882, *P* < 2·2e-16; Figure 2(f)), and fecal metabolites (Spearman’s *ρ* = 0·211, *P* < 2·2e-16; Figure 2(g)), respectively. To determine if these correlations differed by FS status, we fit linear regression models in which each feature (e.g. each species) contributed a stability-*v*-abundance data point, and FS status served as an interaction term. We found a significant interaction for microbial species (*P* = 0·034) such that the slope between stability and abundance differed significantly for FI and VAR over FS status (*Q* ≤ 0·021). In other words, for these groups, an increase in species abundance was associated with either a steeper or shallower increase in stability than what we observed in the FS group. This suggests that FI and variably FS students might rely on certain microbial species to maintain a more consistent community (higher stability) under changing dietary conditions – particularly for those species that are more abundant. A similar pattern emerged for estimated functional pathways (*P* = 0·004), where FI and FS showed a steeper stability–abundance relationship than VAR (*Q* ≤ 0·041). These findings indicate that the more abundant functional pathways remained more stable across time for FI and FS, whereas for VAR, abundance did not translate into stability to the same extent. In contrast, there was no significant difference in faecal metabolome stability by FS classification (*P* = 0·189). Because these analyses are done at the feature level, each reported slope represents an average effect across all species, pathways or metabolites.

### Differential feature shift and abundance in gut microbes, functional pathways and metabolites by food security status

**Figure 3.**
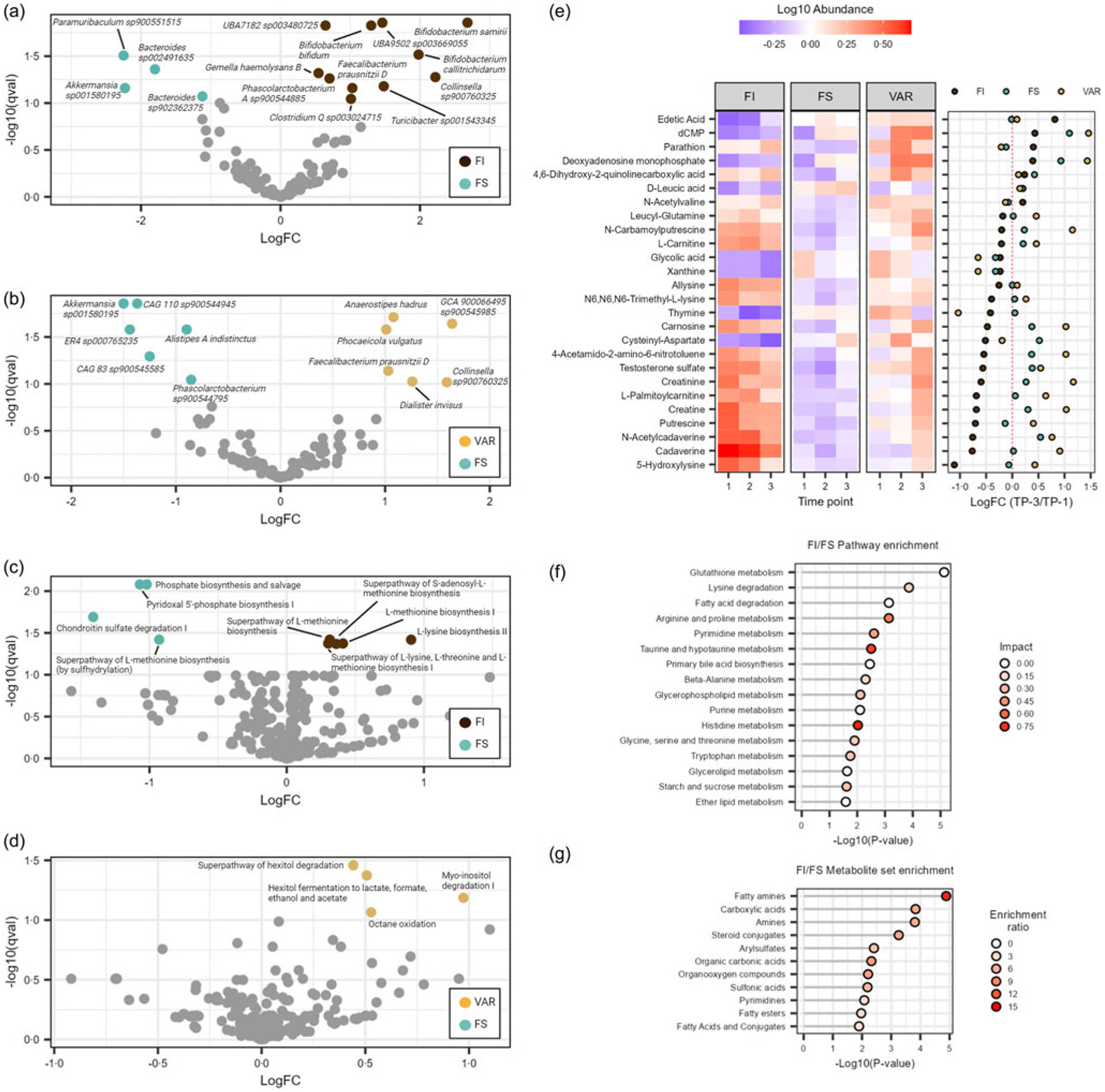
Volcano plots display the differential abundance of significant microbial species for (a) food insecure (FI) and (b) VAR and estimated functional pathways for (c) FI and (d) VAR with FS as the reference group (*Q* < 0·10). Faecal metabolome analysis showing (e) significant between-group metabolites as determined by a general linear model adjusted for time, sex and BMI. Significant metabolites are displayed over the three time periods by log10 abundance and logFC values (ordered by FI positive to negative change) between the first and last sample for each food secure (FS) classification. (f) Significantly altered metabolic pathways and (g) metabolite sets at the main chemical class level comparing FI to FS status (*Q* < 0·10). Note: Pathway impact scores summarise normalised topology measures of differential metabolites in each pathway. The enrichment ratio is computed by number of observed hits/expected number of hits.

To better understand the significant group × time interaction, we examined the differences between the three FS classifications by multivariate regression models, controlling for time, sex and baseline BMI with FS as the reference. We found that the FI group had significantly greater species abundance of *Bifidobacterium samirii, Collinsella sp900760325, Bifidobacterium callitrichidarum, Turicibacter sp001543345, UBA9502 sp003669055* (unclassified *Lachnospiraceae*), *Bifidobacterium bifidum, Faecalibacterium prausnitzii D, Clostridium Q sp003024715, Phascolarctobacterium A sp900544885, UBA7182 sp003480725* (unclassified *Lachnospiraceae*) and *Gemella haemolysans* (log fold-change (logFC): 0·55–2·69, *Q* ≤ 0·090; Figure 3(a)). Conversely, there was a significantly lower abundance in the family *Butyricicoccaceae* (logFC: −1·163, *Q* = 0·097) and species *Paramuribaculum sp900551515, Akkermansia sp001580195, Bacteroides sp002491635* and *Bacteroides sp902362375* in the FI group (logFC: −2·25–1·12, *Q* ≤ 0·008). For VAR participants, we observed greater abundance in *Dialisteraceae* at the family level (logFC: 1·23, *Q* = 0·097) and *GCA 900066495 sp900545985* (unclassified *Peptostreptococcaceae*), *Collinsella sp900760325, Dialister invisus, Anaerostipes hadrus, Phocaeicola vulgatus* and *Faecalibacterium prausnitzii D* at the species level (logFC: 1·01–1·64, *Q* ≤ 0·096; Figure 3(b)). There was a lower abundance of *Akkermansia sp001580195, ER4 sp000765235* (unclassified *Oscillospiraceae*), CAG-110 sp900544945 (unclassified *Oscillo spiraceae*), CAG-83 sp900545585 (unclassified *Oscillospiraceae*), *Alistipes A indistinctus* and *Phascolarctobacterium sp900544795* in the VAR group (logFC: −0·85–1·50, *Q* ≤ 0·090).

At the functional pathway level, examining the differences between FS classification with FS as the reference, we found that the FI group had significantly greater abundance of L-lysine biosynthesis II, L-methionine biosynthesis I, super pathway of S-adenosyl-L-methionine biosynthesis, super pathway of L-lysine, L-threonine and L-methionine biosynthesis I and super pathway of L-methionine biosynthesis (transsulfuration) (logFC: 0·30–0·91, *Q* ≤ 0·043; Figure 3(c)). Conversely, there was a significantly lower abundance in chondroitin sulfate degradation I (bacterial), pyridoxal 5’-phosphate biosynthesis I, phosphate biosynthesis and salvage and super pathway of L-methionine biosynthesis (by sulfhydrylation) (logFC: −1·41–0·93, *Q* ≤ 0·038). For VAR participants, we observed greater pathway abundance in octane oxidation, myo-inositol degradation I, hexitol fermentation to lactate, formate, ethanol and acetate and super pathway of hexitol degradation (bacteria) (logFC: 0·44–0·97, *Q* ≤ 0·086; Figure 3(d)).

To assess potential individual feature shifts in the faecal metabolome, we pooled samples from all time points and assembled a covariate-adjusted general linear model, accounting for time, sex and baseline BMI. With FS status as the reference group, we observed significant differences across classifications on 26 metabolites (*Q* ≤ 0·089). FI samples displayed increases in edetic acid, parathion and N-acetylvaline and decreases in n-arbamoylputrescine, L-carnitine, allysine, N6,N6,N6-trimethyl-L-lysine, thymine, carnosine, cysteinyl-aspartate, 4-aceotamido-2-amino-6-nitroluene, testosterone sulfate, creatinine, L-palmitoylcarnitine, creatine, putrescine, n-acetylcadaverine, cadaverine and 5-hydroxylysine (Figure 3(e)). It should be noted that these metabolites did change over time, though many were depleted (e.g. edetic acid, D-leucic acid, thymine and cysteinyl-aspartate) or enriched in abundance (e.g. cadaverine, N-acetylcadaverine, 5-hydroxylysine and putrescine) as compared to FS participants. Of note, VAR shared trends in abundance change with the other classifications, though unlike either FI or FS metabolite shifts, there was a pattern for increased N-carbamoylputrescine, carnosine, 4-acetamido-2-amino-6-nitrotoluene, creatinine, L-palmitoylcarnitine, creatine and cadaverine (logFC > 0·5).

Next, a PLS-DA model was constructed using levels of significant between-group metabolites solely for FS and FI classifications because of the complexities involved in the status change for VAR. The model accounted for 37·9 % of between-group variance (online Supplementary Figure S2A). Notably, PLS-DA regression coefficients showed that cadaverine, N-acetylcadaverine, putrescine, testosterone sulfate and creatine accounted for the greatest influence of between-group differences (model coefficients ≥ 60·84) and were all weighted towards FI status. Although the PLS-DA model displayed considerable accuracy (*R^2^X* = 0·789), it exhibited low explanatory capacity (*R^2^Y* = 0·308) and poor predictive capacity (*R^2^Q* = 0·172). To assess the robustness of the PLS-DA model, 1000 permutations were performed to confirm the model was not overfit (online Supplementary Figure S2B; observed *P* < 0·001).

Comparing FI to FS, pathway enrichment analysis detected sixteen significant pathways (*Q* ≤ 0·091; Figure 3(f)). The most significant pathways were glutathione metabolism, lysine degradation and fatty acid degradation (*Q* ≤ 0·01), whereas those with the greatest impact included histidine metabolism (I = 0·770), taurine and hypotaurine metabolism (I = 0·714) and arginine and proline metabolism (I = 0·538). At the main chemical class level, enrichment analysis queried against a reference library of 464 metabolite sets revealed enrichment of fatty amines, carboxylic acids, amines, steroid conjugates, arylsulfates, organic carbonic acids, organooxygen compounds, sulfonic acids, pyrimidines, fatty esters and fatty acids and conjugates (FI/FS: *Q* ≤ 0·098; Figure 3(g)). Compared to FS, the faecal metabolome of VAR participants revealed significant enrichment of organic thiophosphoric acids, steroid conjugates, purines and butyrophenones (*Q* ≤ 0·094; data not shown). This was not the case for metabolic pathway enrichment analysis, where no differences were detected (*Q* ≥ 0·329; data not shown).

### Multi-Omic integration suggests enriched food insecure microbes and co-occurrent faecal metabolites influence important metabolic processes in the gut

**Figure 4.**
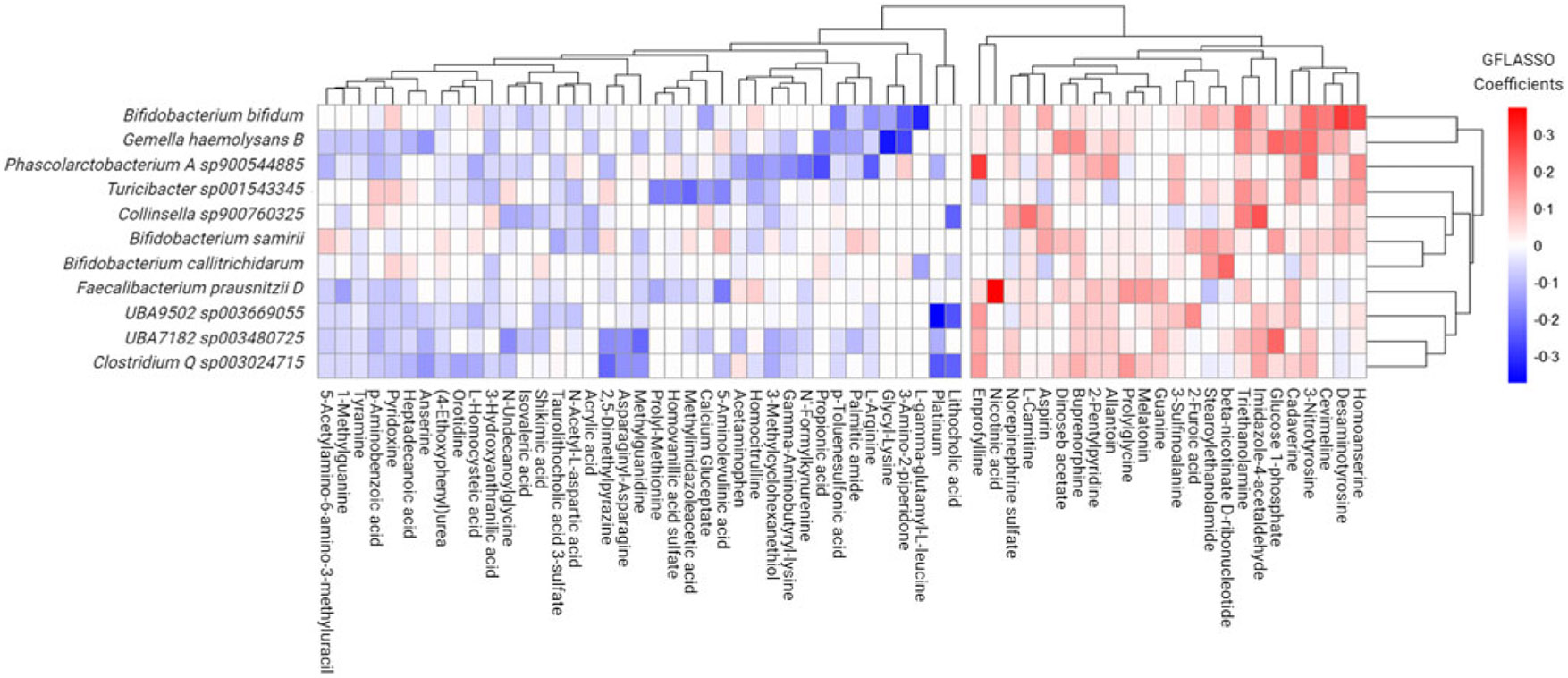
Grid-fused least absolute shrinkage and selection operator (GFLASSO) regression of faecal metabolites that best predicted species abundance of significant microbes associated with food insecure (FI) status.

Seeking to uncover potential multi-omic signatures within the FI microbiome environment, we selected differentially abundant FI species (*v*. FS classification) and the full set of metabolome features channeling these data sets in a regularised high-dimensional regression framework via GFLASSO. This model was selected due to its ability to handle large numbers of predictors and outcomes in comparison to the number of samples. To determine metabolites predictive of co-occurrence with driving features of the FI microbiome, we selected those microbes with a total regression coefficient greater than 0·2 to determine the most prominent signals, as previously described^(42)^. This rendered eleven species sets of sixty-three metabolites each, containing a total of approximately 11 % of the entire reliably detected and annotated faecal metabolome (Figure 4). Metabolites with positive betacoefficients for significantly greater microbial species abundance with FI status were then selected and re-entered into a pathway enrichment analysis to determine how these multi-omic signatures might be influencing metabolism in the GM. Significantly enriched pathways included glutathione metabolism (enrichment ratio (ER): 18·41), lysine degradation (ER: 12·18) and tryptophan metabolism (ER: 4·05) for FI relative to FS (*Q* ≤ 0·091).

Focusing on the most pronounced co-occurrent patterns, we observed notable correlations between specific species and faecal metabolites through the use of Spearman correlations as a complementary approach, bolstering confidence in GFLASSO findings. Noteworthy correlations included: (1) *Bifidobacterium bifidum* with homoanserine (Spearman’s *ρ* = 0·17, *Q* = 0·027), desaminotyrosine (Spearman’s *ρ* = 0·23, *Q* = 1·36e-04), cadaverine (Spearman’s *ρ* = 0·32, *Q* = 7·73e-06), triethanolamine (Spearman’s *ρ* = 0·35, *Q* = 9·25e-07), *γ*-L-Glutamyl-L-cysteine (Spearman’s *ρ* = −0·26, *Q* = 2·21e-04), as well as putrescine (Spearman’s *ρ* = 0·38, *Q* = 1·55e-07) and n-acetylcadaverine (Spearman’s *ρ* = 0·35, *Q* = 9·62e-07); (2) *Gmella haemolysans* with glucose 1-phosphate (Spearman’s *ρ* = 0·33, *Q* = 3·20e-06), cadaverine (Spearman’s *ρ* = 0·26, *Q* = 2·72e-04), n-acetylcadaverine (Spearman’s *ρ* = 0·27, *Q* = 2·05e-04), putrescine (Spearman’s *ρ* = 0·27, *Q* = 1·86e-04), desaminotyrosine (Spearman’s *ρ* = 0·27, *Q* = 1·57e-04) and glycyl lysine (Spearman’s *ρ* = −0·18, *Q* = 0·001); (3) *Phascolarctobacterium A sp900544885* with n-acetylcadaverine (Spearman’s *ρ* = 0·31, *Q* = 1·59e-05) and (4) *Faecalibacterium prausnitzii D* with nicotinic acid (Spearman’s *ρ* = 0·22, *Q* = 0·003).

### Exploration of the gut microbiome during transition from food secure to food insecure status

**Figure 5.**
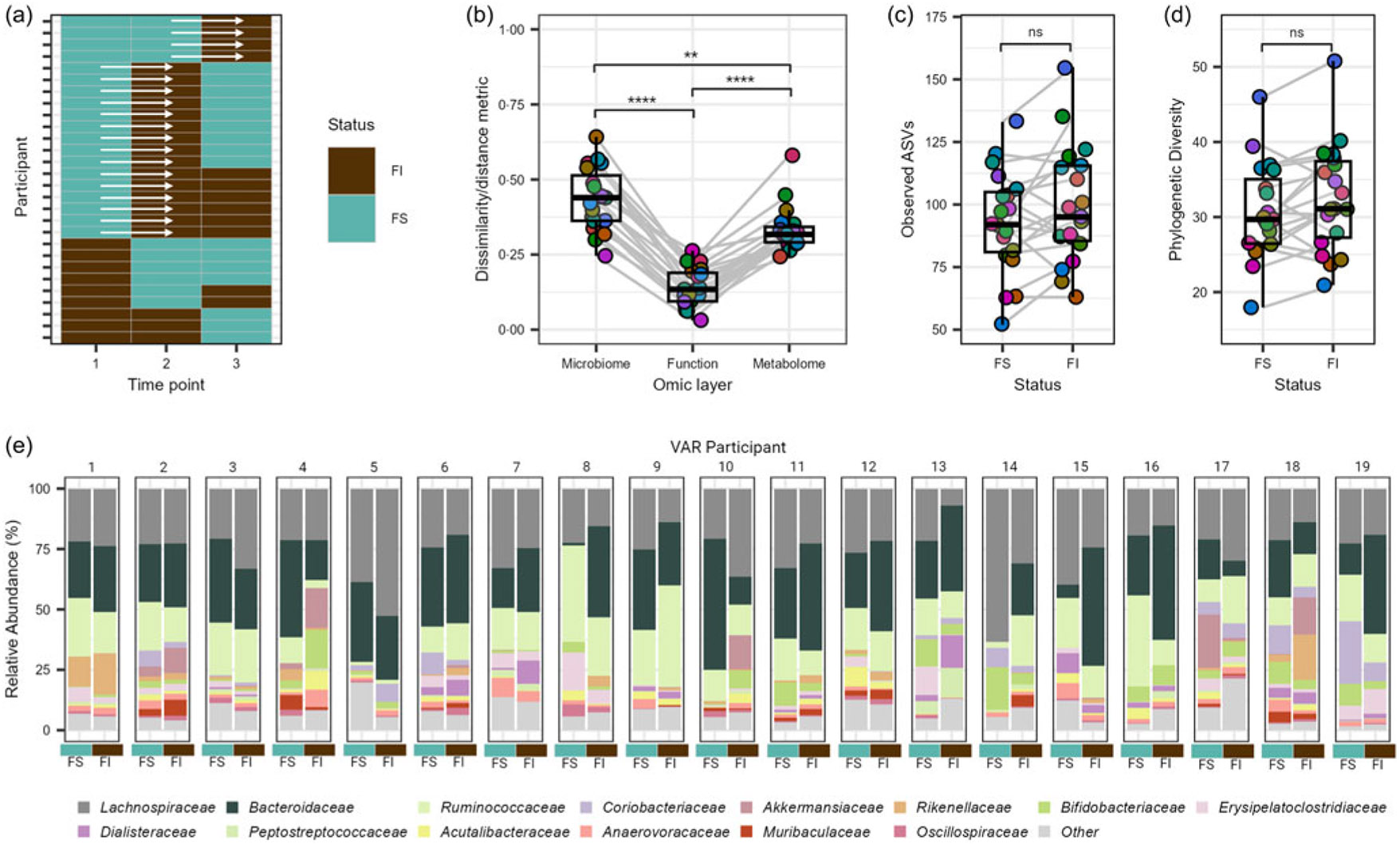
Exploratory analysis of the gut microbiome and metabolome of college students with variable food security (VAR) status. (a) Overview of VAR participant’s transition between food insecure (FI) and food secure (FS) states over the academic year. (b) Intra-individual shifts from FS to FI status in nineteen individuals at the microbial species, estimated functional pathway and faecal metabolome level. Alpha diversity metrics, (c) observed amplicon sequence variants and (d) phylogenetic diversity, capturing the shift from FS to FI status in the nineteen individuals. (e) Relative abundance of the most dominant family level features of the nineteen VAR participants at the FS and FI states. Families with a mean relative abundance of less than 1 % are collapsed in the category ‘Other’. NS = non-significant, ** *P* ≤ 0·01, **** *P* ≤ 0·001.

Exploration of the GM during the transition from FS to FI status was undertaken to gain insights into the dynamic changes that occur under this shift. Leveraging the variability within the VAR group, consisting of twenty-eight individuals, we focused on those who transitioned from initial FS to FI status during the study period, totalling nineteen individuals (Figure 5(a)) and a pre/post sample set of thirty-eight observations for analysis. Assessing intra-individual dissimilarity using Bray–Curtis for the GM, estimated functional pathways and Canberra distance for the faecal metabolome during the transition from FS to FI classification, we observed significant differences across all three omic layers, with the microbial community exhibiting the most pronounced disruption (*Q* ≤ 0·001; Figure 5(b)). While no statistical significance was observed between different FI states for observed ASV and phylogenetic diversity, we noted a trend toward significance for elevated richness and phylogenetic diversity with FI (*P* ≥ 0·369; Figure 5(c)-(d)). To discern significant features during the transition from FS to FI status, we conducted further analyses at the phylum, family and species levels, as well as for estimated functional pathways and faecal metabolites. However, we did not detect any statistically significant differences (*Q* ≥ 0·144). Notably, the observed variability in individual responses and the relatively small sample size within the transition subgroup may have contributed to these results (Figure 5(e)).

## Discussion

In this study, we longitudinally characterised faecal GM composition, estimated function and associated faecal metabolites of 85 dormitory-housed college students with different food security status over the academic year. This approach afforded novel application to an understudied population and deeper phenotyping of the FI microbiome with the combined synergy of microbial sequencing and the faecal metabolome. Overall, our findings indicated a greater overall stability of the GM in college students experiencing persistent or intermittent FI when compared to their FS counterparts. Despite changes in GM taxonomy and faecal metabolite abundance over time, the estimated functional pathways within the GM remained relatively conserved across all groups. Moreover, we observed moderate to large correlations between microbial and estimated functional pathway stability scores and the abundance of individual microbial species. Notably, in a subset of students who transitioned from FS to FI status, we observed significantly greater community-level dissimilarity (Bray–Curtis or Canberra distance) across the GM, predicted functional pathways and faecal metabolome (Q ≤ 0·001); however, no specific phylum-, family- or species-level features or pathways reached statistical significance, and alpha diversity showed only a nonsignificant trend toward higher richness and phylogenetic diversity in FI.

This study delved into the mechanisms through which FI may induce perturbations in GM composition and function. The observed GM stability in individuals experiencing FI may suggest either the conservation of GM function or that the stimuli (e.g. FI assessment within the past 30 days relative to faecal collection) were not potent enough to significantly disrupt GM activity. Previous studies have highlighted the functional redundancy of the human GM, emphasising the highly conserved gene composition across a diverse taxonomic landscape^(43,44)^. Regardless, taxonomic community analysis revealed significant individual variance over time between the three food security classifications at the species level, indicating that food security status influences the composition of gut microbes and serves as a key factor driving changes in the GM. These shifts in microbial composition were confirmed in multivariate regression models, which showed significant differences in species abundance between food security classifications. For instance, *Bifidobacterium samirii, Collinsella sp900760325, Bifidobacterium callitrichidarum* and others were more abundant in the FI group, while *Akkermansia sp001580195* and other species were less abundant. Understanding these differences is not straightforward as microbes positively associated with FI status like *Phocaeicola vulgatus* are context-dependent and may have beneficial (improvement of lipid metabolism^(45)^) or detrimental (involved in human infections^(46)^) influence on the host.

In contrast to microbial composition, estimated functional pathways did not exhibit significant differences between food security classifications in the PERMANOVA analysis. This suggests that, at a functional level, the GM may maintain stability despite changes in microbial species. Nonetheless, examining specific functional pathway trends revealed varying profiles across the food security classifications. For example, FI individuals displayed increased pathways related to energy and microbial turnover, while food-secure individuals showed a broad increase in fatty acid anabolism. These trends are consistent with the presumed health state of the individuals in these different FS classifications, in which FI students may experience erratic or reduced nutrient intake – potentially driving up energy-harvesting processes – while FS students have consistent access to meals, supporting anabolic pathways (e.g. lipid synthesis). However, to pinpoint the exact onset and duration of these functional shifts in the GM, more frequent longitudinal sampling would be necessary. Denser sampling could capture day-to-day or week-to-week changes in diet and microbial function, offering greater insight into how quickly the microbiome responds to fluctuations in food availability.

In the assessment of the faecal metabolome, metabolites that accounted for the most significant differences between food security groups include cadaverine, N-acetylcadaverine, putrescine, testosterone sulfate and creatine, establishing a prominent role in distinguishing FI individuals. Pathway enrichment analysis identified specific metabolic pathways that were significantly enriched in FI individuals compared to FS individuals. These pathways included glutathione metabolism, lysine degradation and fatty acid degradation, suggesting alterations in key metabolic processes associated with food insecurity. Because faecal metabolites can originate from both microbial and host processes, these findings likely reflect microbially mediated metabolic shifts and potential host responses to inconsistent or nutrient-poor food intake. For instance, increases in glutathione-related pathways may indicate heightened oxidative stress handling in the gut environment due to poor nutritional quality and the consumption of energy-dense, nutrient-poor foods^(47)^. This stress could be due to the cyclical nature of food availability, leading to periods of overeating followed by scarcity. In relation, greater lysine and fatty acid degradation could reflect adaptations in microbial and/or host metabolism when carbohydrate or protein availability is limited. This could also be a compensatory mechanism to maintain energy homeostasis when dietary intake is insufficient. Finally, the degradation of fatty acids, or beta-oxidation, is a metabolic process that provides energy when carbohydrate availability is low. In FI individuals, increased fatty acid degradation may suggest reliance on fat stores for energy due to inconsistent access to food, particularly carbohydrates. Although we discuss possible physiological implications for humans experiencing FI (e.g. cyclical intake of nutrient-dense yet low-quality foods), our data do not definitively isolate whether these catabolic processes are predominantly driven by host *v* microbial mechanisms. Future studies employing direct host biomarkers (e.g. blood metabolites) or shotgun metagenomic/metatranscriptomic approaches would help clarify the specific contributions of the GM relative to the host under conditions of food insecurity.

To uncover potential multi-omic signatures within the FI microbiome, a high-dimensional regression framework was applied, identifying specific metabolites associated with FI microbial species. Correlation analyses confirmed co-occurring patterns between certain microbial species and faecal metabolites. Notable positive correlations included those between *Bifidobacterium bifidum* and cadaverine, triethanolamine, putrescine and n-acetylcadaverine. Similarly, *Gemella haemolysans* was positively associated with cadaverine, putrescine and n-acetylcadaverine, as well as *Phascolarctobacterium A sp900544885* with n-acetylcadaverine and F*aecalibacterium prausnitzii D* with nicotinic acid. The current understanding of these specific metabolites is largely underdeveloped with respect to their overall presence, physiological role, functional pathways and effects on the human body. Despite the paucity of studies acknowledging exogenous (i.e. food sources) and endogenous (i.e. cell synthesis) sources of these polyamines, putrescine or cadaverine are thought to be more greatly produced from microbial sources^(48)^; a connection that is still relatively uncharacterised. Very few studies to date have accomplished reporting linkages between metabolite production and GM composition as demonstrated in this study^(49)^. For example, Kitada *et al.* discovered novel linkages between three commensal bacterial populations, including *Bifidobacterium spp*. and the enhanced production of putrescine^(50)^, a polyamine commonly found in the intestinal lumen within the human gut as well as in a variety of foods and food products, with higher concentrations typically found in fermented dairy products and animal-based processed foods^(51)^. Interestingly, soluble and fermentable dietary fibre consumption has been shown to enhance the gut microbial production of PAs putrescine, cadaverine and spermidine in rodents with gut microbes playing a key role^(52-54)^. Previous research suggests the importance of PAs from dietary constituents for meeting the needs of the human body ^((55)^ which support intestinal DNA, RNA and protein synthesis, as well as cellular proliferation and differentiation^(56,57)^. Pathway enrichment analyses further support this idea in that gut microbes are capable of decarboxylating lysine to cadaverine, and lysine degradation was identified as a key pathway in FI participants. Overall, these data suggest that microbially-derived PAs may serve as energy substrates^(58)^ in the gut during periods of FI or intermittent food access.

Based on the findings above, we can tangentially begin to make connections to how these interactions may be reflected within the human GM in response to food insecurity. For example, recent evidence reports in extreme FI conditions, such as migration/forced displacement, that certain coping strategies may develop in order to satisfy hunger or to ensure other members of the household are receiving adequate nutrition (e.g. children, elderly). One of these coping strategies includes consuming expired or contaminated food products, which may lead to the increased production, circulation and excretion of polyamine from the human body due to increased microbial activity^(59,60)^. Finally, the positive correlation observed in this study between *F. prausnitzii D* and nicotinic acid (vitamin B_3_) is intriguing. While several microbial species obtain the vitamin B_3_ biosynthesis pathway, the role of vitamin B_3_ in the human GM is still considered relatively underdeveloped. Specifically investigating this relationship between *F. prausnitzii D* and vitamin B_3_, we can again turn to other evidence to help support this potential phenomenon. For example, studies have confirmed that the butyrate-producer *F. prausnitzii* increases with enhanced uptake of riboflavin (vitamin B_2_)^(61,62)^. Vitamin B_3_ can be synthesised from the amino acid, tryptophan, where vitamin B_2_ serves as an important cofactor in relation to energy metabolism. While these two B vitamins share similar food sources, such as meat, dairy, eggs and fortified foods, this positive correlation between vitamin B_3_ and *F. prausnitizii D* may reveal an underlying mechanism not yet well understood that warrants further investigation^(63)^ within the specific context of food insecurity and impacts on the human condition.

It is essential to recognise that FI extends beyond dietary factors and encompasses important social and psychological dimensions, such as psychological well-being and stress, which can influence the relationship between FI and dietary intake^(64)^. These factors may underpin the complex interplay between FI and GM structure and function^(18)^. Our previous research has highlighted the influence of these psychological factors in the context of FI. For example, we reported FI students had elevations in the faecal metabolites, picolinic acid, 2-pyrrolidinone and phosphocreatine^(18)^. These metabolites are central to neurological health^(65)^, gut axis signalling^(66)^ and energy metabolism^(67)^, respectively. Many of the microbial and metabolomic features detected in the present analysis seem to be more nutritionally orientated, though could have a secondary influence in relation to the gut–brain axis. Future work will be required with expanded longitudinal sampling as mentioned previously.

Very few studies have explored associations between FI and GM composition. An impactful strength of the present work was the longitudinal nature of the data allowing for the study of persistent and intermittent periods of FI in relation to the GM and metabolome and estimated functional activities. To our knowledge, no studies have explored the longitudinal relationship between FI and GM structure in a vulnerable adolescent population. Although our sample size of eighty-five participants may be considered moderate for an observational study, the repeated measures and multi–omic data still provided meaningful insights into the complex interplay of social, biological and physiological factors underlying FI. Lastly, stool samples serve as a valuable source of objective biological data reflecting GM–host interactions, complementing self-reported dietary measures in understanding the holistic dietary and physiological context.

Despite the mentioned strengths, this study was not without limitations. Firstly, there was a sample size difference between the FI and FS groups that occurred as a result of investigating a convenience sample. Although not balanced, these groups were representative of the student population under study^(68,69)^ and cohorts from observational studies of food security among college students^(3,18)^. Further, although eighty-five participants provided sufficient longitudinal data to detect notable differences, this is considered moderate for an observational study; subgroup analyses with fewer than thirty individuals inherently limit the detection of smaller effect sizes. Secondly, while dietary intake was assessed using the National Cancer Institute Dietary Screener Questionnaire, these data were not used as covariates because they did not significantly differ across the food security groups. Moreover, the screener does not capture the variety of foods consumed or potentially relevant eating behaviours (e.g. intermittent fasting), making it less sensitive in detecting nuanced dietary patterns that could affect the GM. Consequently, we could not fully capture differences in dietary diversity between groups. It is possible that FS participants had access to a wider variety of foods, contributing to the greater variability in their GM observed in this study. Thirdly, another limitation of this work was the lack of validated instruments for assessing FI in college students^(70)^, as the instrument used has only been validated in low-income adults^(70)^. Albeit, most studies of college students use a version of the measure used in the current study^(27)^. All of the students in this study had purchased a pre-paid meal plan, which in theory minimises food insecurity. However, our prior research has shown that even students who pre-paid for an unlimited meal plan report food insecurity^(5)^. Potential explanations for this apparently contradictory finding include conflicts with students’ schedules and dining hall hours and student time management. Fourth, although our multi-omic design provides novel insights, we inferred microbial function using 16S rRNA gene sequencing rather than employing shotgun metagenomic approaches. Because reference databases for 16S-based functional prediction are incomplete, these functional inferences could introduce biases and must be interpreted cautiously. Finally, while we characterised overall relationships between abundance and stability, we did not define a ‘core’ or exceptionally stable set of taxa or functions, as this would require threshold-specific analyses and multiple comparisons with more frequent sampling that extend beyond the scope of the present study.

In summary, the results suggest that food security status is associated with significant shifts in the gut microbial composition, metabolite profiles and metabolic pathways among college students. These findings underscore the complex interplay between food security, the GM and host metabolism, highlighting the potential role of the GM in responding to dietary and environmental factors associated with food insecurity. Moving forward, future research should focus on addressing the limitations identified, such as employing more robust measures for assessing food security, dietary intake and mental health symptoms associated with food insecurity. Additionally, diversifying the study population to include individuals not bound by dorm contracts with provided food could offer valuable insights into the broader spectrum of food security experiences among college students. Further investigation is needed to elucidate the mechanistic links between these observations and their implications for health and well-being in this population.

## Supplementary Material

1

**Supplementary material.** For supplementary material/s referred to in this article, please visit https://doi.org/10.1017/S0007114525103668

## Figures and Tables

**Table 1.** Baseline demographic, anthropometric and behavioural characteristics of study participants (Mean values and standard deviations; numbers and percentages)

Characteristic	Total (*n* 85)		FI (*n* 13)		FS (*n* 44)		VAR (*n* 28)	
			
	Mean	sd	Mean	sd	Mean	sd	Mean	sd
Age (years)	18·6	0·8	18·5	0·5	18·7	0·9	18·6	0·6
	%	*n*	%	*n*	%	*n*	%	*n*
Sex, % (*n*)								
Male	38·8	33	53·8	7	45·5	20	21·4	6
Female	61·2	52	46·2	6	54·5	24	78·6	22
Race/ethnicity % (*n*)								
White	49·4	42	30·8	4	56·8	25	46·4	13
Black	11·8	10	15·4	2	11·4	5	10·7	3
Hispanic	21·2	18	23·1	3	20·5	9	21·4	6
Other	17·6	15	30·8·4	4	11·4	5	21·4	6
	Mean	sd	Mean	sd	Mean	sd	Mean	sd
Height (cm)	168·6	10·5	168·1	12·8	170·3	9·6	166·5	10·8
Weight (kg)	71·1	17·7	63·4	13·3	73·1	16·9	71·2	19·9
Waist circumference (cm)	82·4	13·5	75·9	7·4	84·1	13·5	82·6	14·9
Waist/hip ratio	0·82	0·07	0·78	0·05	0·83	0·07	0·82	0·07
BMI (kg/m^2^)	24·8	5·1	22·2	2·1	25·1	4·9	25·5	6·1
	%	*n*	%	*n*	%	*n*	%	*n*
BMI categories (kg/m^2^), % (*n*)								
< 18·5 kg/m^2^	5·9	5	7·7	1	4·5	2	7·1	2
18·5–24·9 kg/m^2^	57·6	49	84·6	11	50·0	22	57·1	16
25–29·9 kg/m^2^	24·7	21	7·7	1	31·8	14	21·4	6
≥ 30 kg/m^2^	11·8	10	–		13·6	6	14·3	4
	Mean	sd	Mean	sd	Mean	sd	Mean	sd
MVPA (min/d)	48·2	31·1	56·9	33·7	46·7	30·7	46·8	31·2
Stress score	8·0	2·3	8·9	1·8	7·6	2·4	8·2	2·2
Depression score	1·9	0·8	2·3	0·7*	1·7	0·7	2·1	0·8
Hours of nightly sleep^†^	7·4	0·9	6·8	1·3	7·6	0·9	7·4	0·9
Alcohol intake^‡^	3·2	5·5	7·6	10·0*	1·8	2·9	3·7	5·1
Fruit/vegetable intake^§^	2·5	0·8	2·5	0·8	2·7	0·9	2·2	0·7
Whole grain intake^∥^	0·8	0·3	0·7	0·4	0·8	0·3	0·7	0·3
Dairy intake^§^	2·0	0·9	2·1	0·9	2·2	0·9	1·8	0·6
Red/processed meat intake^∥^	0·9	0·8	0·9	0·8	0·9	0·8	0·9	0·7
Daily sugar intake (g)	19·1	7·2	21·5	8·5	17·7	5·8	20·3	8·3
Daily fibre intake (g)	16·4	3·6	16·8	4·2	17·1	3·5	15·0	3·3

Data displayed as mean (sd), unless stated otherwise.

Abbreviations: FI, food insecure; FS, food secure; VAR, food variable; MVPA, moderate-to-vigorous physical activity.

* Significant difference between FI and FS (pairwise comparison), *P* < 0·05.

† Average of combined weekday and weekend nightly sleep hours.

‡ Number of beverages over the last 7 d.

§ Expressed as daily cup equivalents.

∥ Daily number of servings.

## Abbreviations:

ASV: amplicon sequence variants
ER: enrichment ratio
FI: food insecure
FS: food secure
GFLASSO: graph-guided fused least absolute shrinkage and selection operator
GM: gut microbiome
MS: exact mass
PLS-DA: partial least squares-discriminant analysis
SPARC: Social impact of Physical Activity and nutRition in College
VAR: variable food secure

## References

[1] Department of Agriculture Economic Research Service (2023) Household Food Security in the United States in 2022. 10.32747/2023.8134351.ers (accessed February 2024).

[2] Coleman-Jensen A, Gregory C & Singh A (2014) Household food security in the United States in 2013. SSRN Electron J. 10.2139/ssrn.2504067.

[3] Bruening M, Argo K, Payne-Sturges D, (2017) The struggle is real: a systematic review of food insecurity on postsecondary education campuses. J Acad Nutr Diet 117, 1767–1791. 28754200 10.1016/j.jand.2017.05.022 PMC6901286

[4] Larson N, Laska MN & Neumark-Sztainer D (2020) Food insecurity, diet quality, home food availability, and health risk behaviors among emerging adults: findings from the EAT 2010–2018 Study. Am J Public Health 110, 1422–1428. 32673120 10.2105/AJPH.2020.305783 PMC7427214

[5] van Woerden I, Hruschka D, Vega-López S, (2019) Food insecure college students and objective measurements of their unused meal plans. Nutrients 11, 904. 31018554 10.3390/nu11040904 PMC6521619

[6] Bruening M, Brennhofer S, van Woerden I, (2016) Factors related to the high rates of food insecurity among diverse, urban college freshmen. J Acad Nutr Diet 116, 1450–1457. 27212147 10.1016/j.jand.2016.04.004 PMC5520984

[7] Woerden I, Hruschka D & Bruening M (2019) Food insecurity negatively impacts academic performance. J Public Aff 19, e1864.

[8] Gundersen C & Ziliak JP (2017) Food insecurity and health outcomes. Health Affair 34, 1830–1839. 10.1377/hlthaff.2015.0645 26526240

[9] Kendall A, Olson CM & Frongillo EA (1996) Relationship of hunger and food insecurity to food availability and consumption. J Am Diet Assoc 96, 1019–1024. 8841164 10.1016/S0002-8223(96)00271-4

[10] Hazzard VM, Loth KA, Hooper L, (2020) Food insecurity and eating disorders: a review of emerging evidence. Curr Psychiat Rep 22, 74. 10.1007/s11920-020-01200-0 PMC7596309 33125614

[11] Stinson EJ, Votruba SB, Venti C, (2018) Food insecurity is associated with maladaptive eating behaviors and objectively measured overeating. Obesity 26, 1841–1848. 30426695 10.1002/oby.22305 PMC6249092

[12] Nagata JM, Palar K, Gooding HC, (2019) Food insecurity is associated with poorer mental health and sleep outcomes in young adults. J Adolesc Heal 65, 805–811. 10.1016/j.jadohealth.2019.08.010 PMC6874757 31587956

[13] Mohr AE, Jasbi P, Bowes DA, (2022) Exploratory analysis of one *v.* two-day intermittent fasting protocols on the gut microbiome and plasma metabolome in adults with overweight/obesity. Front Nutr 9, 1036080. 36386914 10.3389/fnut.2022.1036080 PMC9644216

[14] von Schwartzenberg RJ, Bisanz JE, Lyalina S, (2021) Caloric restriction disrupts the microbiota and colonization resistance. Nature 595, 272–277. 34163067 10.1038/s41586-021-03663-4 PMC8959578

[15] Fan Y, Støving RK, Ibraim SB, (2023) The gut microbiota contributes to the pathogenesis of anorexia nervosa in humans and mice. Nat Microbiol 8, 787–802. 37069399 10.1038/s41564-023-01355-5 PMC10159860

[16] Mohr AE, Sweazea KL, Bowes DA, (2024) Gut microbiome remodeling and metabolomic profile improves in response to protein pacing with intermittent fasting *v.* continuous caloric restriction. Nat Commun 15, 4155. 38806467 10.1038/s41467-024-48355-5 PMC11133430

[17] Vila AV, Hu S, Andreu-Sánchez S, (2023) Faecal metabolome and its determinants in inflammatory bowel disease. Gut 72, 1472–1485. 36958817 10.1136/gutjnl-2022-328048 PMC10359577

[18] Mohr AE, Jasbi P, Wyst KBV, (2022) Association of food insecurity on gut microbiome and metabolome profiles in a diverse college-based sample. Sci Rep-UK 12, 14358. 10.1038/s41598-022-18515-y PMC9399224 35999348

[19] Mehta RS, Abu-Ali GS, Drew DA, (2018) Stability of the human faecal microbiome in a cohort of adult men. Nat Microbiol 3, 347–355. 29335554 10.1038/s41564-017-0096-0 PMC6016839

[20] Bruening M, Ohri-Vachaspati P, Brewis A, (2016) Longitudinal social networks impacts on weight and weight-related behaviors assessed using mobile-based ecological momentary assessments: study protocols for the SPARC study. BMC Public Health 16, 901. 27576358 10.1186/s12889-016-3536-5 PMC5006372

[21] Sirard JR & Pate RR (2001) Physical activity assessment in children and adolescents. Sports Med 31, 439–454. 11394563 10.2165/00007256-200131060-00004

[22] Utter J, Neumark-Sztainer D, Jeffery R, (2003) Couch potatoes or French fries: are sedentary behaviors associated with body mass index, physical activity, and dietary behaviors among adolescents? J Am Diet Assoc 103, 1298–1305. 14520247 10.1016/s0002-8223(03)01079-4

[23] National Cancer Institute (2009) Dietary Screener Questionnaire in the NHANES 2009–2010. https://appliedresearch.cancer.gov/nhanes/dietscreen/dsq_english.pdf (accessed June 2022).

[24] American College Health Association (2013) American College Health Association-National College Health Assessment II: Reference Group Report Spring 2013. Hanover, MD: American College Health Association.

[25] Cohen S, Kamarck T & Mermelstein R (1983) A global measure of perceived stress. J Heal Soc Behav 24, 385–396. 6668417

[26] U.S. Household Food Security Survey Module: Three-Stage Design, With Screeners (2012). https://www.ers.usda.gov/topics/food-nutrition-assistance/food-security-in-the-us/survey-tools/#household (accessed March 2023).

[27] Nikolaus CJ, Ellison B & Nickols-Richardson SM (2019) Are estimates of food insecurity among college students accurate? Comparison of assessment protocols. Plos One 14, e0215161. 31017912 10.1371/journal.pone.0215161 PMC6481800

[28] Jasbi P, Mohr AE, Shi X, (2022) Microbiome and metabolome profiles of high screen time in a cohort of healthy college students. Sci Rep-UK 12, 3452. 10.1038/s41598-022-07381-3 PMC8891328 35236903

[29] Bolyen E, Rideout JR, Dillon MR, (2019) Reproducible, interactive, scalable and extensible microbiome data science using QIIME 2. Nat Biotechnol 37, 852–857. 31341288 10.1038/s41587-019-0209-9 PMC7015180

[30] Chaumeil P-A, Mussig AJ, Hugenholtz P, (2022) GTDB-Tk v2: memory friendly classification with the genome taxonomy database. Bioinf 38, 5315–5316. 10.1093/bioinformatics/btac672 PMC9710552 36218463

[31] Kaehler BD, Bokulich NA, McDonald D, (2019) Species abundance information improves sequence taxonomy classification accuracy. Nat Commun 10, 4643. 31604942 10.1038/s41467-019-12669-6 PMC6789115

[32] McKnight DT, Huerlimann R, Bower DS, (2019) Methods for normalizing microbiome data: an ecological perspective. Methods Ecol Evol 10, 389–400.

[33] Douglas GM, Maffei VJ, Zaneveld JR, (2020) PICRUSt2 for prediction of metagenome functions. Nat Biotechnol 38, 685–688. 32483366 10.1038/s41587-020-0548-6 PMC7365738

[34] Caspi R, Billington R, Ferrer L, (2016) The MetaCyc database of metabolic pathways and enzymes and the BioCyc collection of pathway/genome databases. Nucleic Acids Res 44, D471–D480. 26527732 10.1093/nar/gkv1164 PMC4702838

[35] Scieszka DP, Garland D, Hunter R, (2023) Multi-omic assessment shows dysregulation of pulmonary and systemic immunity to e-cigarette exposure. Respir Res 24, 138. 37231407 10.1186/s12931-023-02441-2 PMC10209577

[36] Wishart DS, Guo A, Oler E, (2021) HMDB 5.0: the human metabolome database for 2022. Nucleic Acids Res 50, D622–D631. 10.1093/nar/gkab1062 PMC8728138 34986597

[37] Raghuvanshi R, Vasco K, Vázquez-Baeza Y, (2020) High-resolution longitudinal dynamics of the cystic fibrosis sputum microbiome and metabolome through antibiotic therapy. Msystems 5, e00292–20. 32576651 10.1128/mSystems.00292-20 PMC7311317

[38] Faith JJ, Guruge JL, Charbonneau M, (2013) The long-term stability of the human gut microbiota. Science 341, 1237439. 23828941 10.1126/science.1237439 PMC3791589

[39] Mallick H, Rahnavard A, McIver LJ, (2021) Multivariable association discovery in population-scale meta-omics studies. Plos Comput Biol 17, e1009442. 34784344 10.1371/journal.pcbi.1009442 PMC8714082

[40] Pang Z, Zhou G, Ewald J, (2022) Using MetaboAnalyst 5.0 for LC–HRMS spectra processing, multi-omics integration and covariate adjustment of global metabolomics data. Nat Protoc 17, 1735–1761. 35715522 10.1038/s41596-022-00710-w

[41] Huttenhower C, Gevers D, Knight R, (2012) Structure, function and diversity of the healthy human microbiome. Nature 486, 207–214. 22699609 10.1038/nature11234 PMC3564958

[42] Lima FA, Li K, Wen W, (2018) Unraveling lipid metabolism in maize with time-resolved multi-omics data. Plant J 93, 1102–1115. 29385634 10.1111/tpj.13833

[43] Tian L, Wang X-W, Wu A-K, (2020) Deciphering functional redundancy in the human microbiome. Nat Commun 11, 6217. 33277504 10.1038/s41467-020-19940-1 PMC7719190

[44] Li L, Wang T, Ning Z, (2023) Revealing proteome-level functional redundancy in the human gut microbiome using ultra-deep metaproteomics. Nat Commun 14, 3428. 37301875 10.1038/s41467-023-39149-2 PMC10257714

[45] Xu M, Lan R, Qiao L, (2023) Bacteroides vulgatus ameliorates lipid metabolic disorders and modulates gut microbial composition in hyperlipidemic rats. Microbiol Spectr 11, e02517–22. 36625637 10.1128/spectrum.02517-22 PMC9927244

[46] Vu H, Muto Y, Hayashi M, Noguchi H, (2022) Complete genome sequences of three Phocaeicola Vulgatus strains isolated from a healthy Japanese individual. Microbiol Resour Announc 11, e01124–21. 35112912 10.1128/mra.01124-21 PMC8812301

[47] Laraia BA (2013) Food insecurity and chronic disease. Adv Nutr 4, 203–212. 23493536 10.3945/an.112.003277 PMC3649100

[48] Muñoz-Esparza NC, Latorre-Moratalla ML, Comas-Basté O, (2019) Polyamines in Food. Front Nutr 6, 108. 31355206 10.3389/fnut.2019.00108 PMC6637774

[49] Tofalo R, Cocchi S & Suzzi G (2019) Polyamines and gut microbiota. Front Nutr 6, 16. 30859104 10.3389/fnut.2019.00016 PMC6397830

[50] Kitada Y, Muramatsu K, Toju H, (2018) Bioactive polyamine production by a novel hybrid system comprising multiple indigenous gut bacterial strategies. Sci Adv 4, eaat0062. 29963630 10.1126/sciadv.aat0062 PMC6021145

[51] del Rio B, Fernandez M, Redruello B, (2024) New insights into the toxicological effects of dietary biogenic amines. Food Chem 435, 137558. 37783126 10.1016/j.foodchem.2023.137558

[52] Deloyer P, Dandrifosse G, Kok N, (2000) Dietary fructans modulate polyamine concentration in the cecum of rats. J Nutr 130, 2456–2460. 11015472 10.1093/jn/130.10.2456

[53] Noack J, Dongowski G, Hartmann L, (2000) The human gut bacteria Bacteroides thetaiotaomicron and Fusobacterium varium produce putrescine and spermidine in cecum of pectin-fed gnotobiotic rats. J Nutr 130, 1225–1231. 10801923 10.1093/jn/130.5.1225

[54] Noack J, Kleessen B, Proll J, (1998) Dietary guar gum and pectin stimulate intestinal microbial polyamine synthesis in rats. J Nutr 128, 1385–1391. 9687560 10.1093/jn/128.8.1385

[55] Larqué E, Sabater-Molina M & Zamora S (2007) Biological significance of dietary polyamines. Nutrition 23, 87–95. 17113752 10.1016/j.nut.2006.09.006

[56] Ginty DD, Osborne DL & Seidel ER (1989) Putrescine stimulates DNA synthesis in intestinal epithelial cells. Am J Physiol-Gastrointest Liver Physiol 257, G145–G150. 10.1152/ajpgi.1989.257.1.G145 2473652

[57] McCormack SA & Johnson LR (1991) Role of polyamines in gastrointestinal mucosal growth. Am J Physiol-Gastrointest Liver Physiol 260, G795–G806. 10.1152/ajpgi.1991.260.6.G795 2058669

[58] Bardócz S, Grant G, Brown DS, (1998) Putrescine as a source of instant energy in the small intestine of the rat. Gut 42, 24. 9505881 10.1136/gut.42.1.24 PMC1726969

[59] Deschak CI, Infante C, Mundo-Rosas V, (2022) Food insecurity and coping strategies in international migrants in transit through Mexico. J Migr Heal 5, 100099. 10.1016/j.jmh.2022.100099 PMC9019261 35465452

[60] Martínez-Martínez OA, Gil-Vasquez K & Romero-González MB (2023) Food insecurity and levels of marginalization: food accessibility, consumption and concern in Mexico. Int J Equity Heal 22, 178. 10.1186/s12939-023-01977-5 PMC10478370 37667336

[61] Khan MT, Duncan SH, Stams AJM, (2012) The gut anaerobe Faecalibacterium prausnitzii uses an extracellular electron shuttle to grow at oxic–anoxic interphases. ISME J 6, 1578–1585. 22357539 10.1038/ismej.2012.5 PMC3400418

[62] Khan MT, Browne WR, van Dijl JM, (2012) How can Faecalibacterium prausnitzii employ riboflavin for extracellular electron transfer? Antioxid Redox Signal 17, 1433–1440. 22607129 10.1089/ars.2012.4701

[63] Wan Z, Zheng J, Zhu Z, (2022) Intermediate role of gut microbiota in vitamin B nutrition and its influences on human health. Front Nutr 9, 1031502. 36583209 10.3389/fnut.2022.1031502 PMC9792504

[64] Cedillo YE, Kelly T, Davis E, (2023) Evaluation of food security status, psychological well-being, and stress on BMI and diet-related behaviors among a sample of college students. Public Heal 224, 32–40. 10.1016/j.puhe.2023.08.015 37708714

[65] Davis I & Liu A (2015) What is the tryptophan kynurenine pathway and why is it important to neurotherapeutics? Expert Rev Neurother 15, 719–721. 26004930 10.1586/14737175.2015.1049999 PMC4482796

[66] Baj A, Moro E, Bistoletti M, (2019) Glutamatergic signaling along the microbiota-gut-brain axis. Int J Mol Sci 20, 1482. 30934533 10.3390/ijms20061482 PMC6471396

[67] Christ M, Zange J, Janson CP, (2001) Hypoxia modulates rapid effects of aldosterone on oxidative metabolism in human calf muscle. J Endocrinol Investig 24, 587–597. 11686541 10.1007/BF03343899

[68] Bruening M, van Woerden I, Schaefer DR, (2018) Friendship as a social mechanism influencing body mass index (BMI) among emerging adults. Plos One 13, e0208894. 30562375 10.1371/journal.pone.0208894 PMC6298660

[69] Bruening M, van Woerden I, Todd M, (2018) Hungry to learn: the prevalence and effects of food insecurity on health behaviors and outcomes over time among a diverse sample of university freshmen. Int J Behav Nutr Phy 15, 9. 10.1186/s12966-018-0647-7 PMC5774124 29347963

[70] Ellison B, Bruening M, Hruschka DJ, (2021) Viewpoint: food insecurity among college students: a case for consistent and comparable measurement. Food Policy 101, 102031.

