# Supplementary material for: Longitudinal assessment of food insecurity status on the gut microbiome and metabolome of first-year college students
Source: Br J Nutr. 2025 Jun 17;133(12):1473–86. doi: 10.1017/S0007114525103668 (PMC12334067; doi:10.1017/S0007114525103668)
Supplement: Mohr et al. supplementary material [file S0007114525103668sup001.docx]

**Supplementary Material**

**Longitudinal assessment of food insecurity status on the gut microbiome and metabolome of first year college students**

A.E. Mohr^1,2^, P. Jasbi^3^, I. van Woerden^4^, D.A. Bowes^5^, J. Chi^1,6^, H. Gu^1,6^, M. Bruening^7^*, C.M. Whisner^1,2^*

^1^College of Health Solutions, Arizona State University, Phoenix, AZ, USA

^2^Center for Health Through Microbiomes, Biodesign Institute, Arizona State University, Tempe, AZ, USA

^3^Systems Precision Engineering and Advanced Research (SPEAR), Theriome Inc., Phoenix, AZ, USA

^4^Department of Community and Public Health, Idaho State University, Pocatello, ID, USA

^5^Department of Environmental Health Sciences, Arnold School of Public Health, University of South Carolina, 921 Assembly Street, Columbia, SC 29208, USA.

^6^Center for Translational Science, Florida International University, Port St. Lucie, FL, USA

^7^Department of Nutritional Sciences, College of Health and Human Development, Pennsylvania State University, University Park, PA, USA

*Corresponding Author

Email: [mmb203@psu.edu](mailto:mmb203@psu.edu)

Email: [cwhisner@asu.edu](mailto:cwhisner@asu.edu)

**Page S-2, Table S1.** Anthropometric, and behavioral characteristics of study participants over time by longitudinal food security status.

**Page S-3, Fig. S1.** Alpha diversity metrics (A) observed amplicon sequence variants and (B) phylogenetic diversity at each of the three sample collection points.

**Page S-4, Fig. S2.** A Partial Least Squares-Discriminant Analysis (PLS-DA) between food insecure (FI) and food secure (FS) individuals using all 26 significant metabolites determined by covariate-controlled general linear modeling. (A) PLS-DA scores plot shows 37.9% of accounted variance between study groups (*R^2^X* = 0.789, *R^2^Y* = 0.308, *R^2^Q* = 0.172). (B) Permutation testing by 1000 iterations shows acceptable model fit (observed *P* < 0.001).

| Table S1. Anthropometric and behavioral characteristics of study participants over time by longitudinal food security status. | | | | | | | | | |
| --- | --- | --- | --- | --- | --- | --- | --- | --- | --- |
|  | **FI (*n*=13)** | | | **FS (*n*=44)** | | | **Variable (*n*=28)** | | |
| Characteristic | **Time 1** | **Time 2** | **Time 3** | **Time 1** | **Time 2** | **Time 3** | **Time 1** | **Time 2** | **Time 3** |
| Weight (kg) | 63.4 ± 13.3 | 64.5 ± 13.8 | 63.7 ± 12.4 | 73.1 ± 16.9 | 74.8 ± 16.7 | 75.0 ± 17.3 | 71.2 ± 19.9 | 74.1 ± 21.2 | 73.5 ± 20.8 |
| Waist circumference (cm) | 75.9 ± 7.4 | 76.6 ± 7.7 | 77.1 ± 6.9 | 84.1 ± 13.5 | 86.0 ± 14.5 | 85.3 ± 13.7 | 82.6 ± 14.9 | 85.1 ± 15.9 | 86.0 ± 15.6 |
| Waist/hip ratio | 0.78 ± 0.05 | 0.80 ± 0.05 | 0.79 ± 0.04 | 0.83 ± 0.07 | 0.85 ± 0.07 | 0.84 ± 0.06 | 0.82 ± 0.07 | 0.84 ± 0.07 | 0.85 ± 0.07 |
| BMI (kg/m^2^) | 22.2 ± 2.1 | 22.9 ± 2.1 | 22.8 ± 2.2 | 25.1 ± 4.9 | 25.6 ± 4.9 | 25.7 ± 5.1 | 25.5 ± 6.1 | 26.5 ± 6.5 | 26.3 ± 6.3 |
| MVPA (min/day) | 56.9 ± 33.7 | 59.5 ± 30.9 | 54.5 ± 33.7 | 46.7 ± 30.7 | 41.3 ± 29.2 | 35.1 ± 26.3 | 46.8 ± 31.2 | 40.8 ± 28.5 | 49.1 ± 32.2 |
| Stress score | 8.9 ± 1.8 | 8.9 ± 2.1 | 8.5 ± 2.6 | 7.6 ± 2.4 | 7.9 ± 2.6 | 7.6 ± 2.3 | 8.2 ± 2.2 | 9.1 ± 2.6 | 9.3 ± 2.7 |
| Depression score | 2.3 ± 0.7* | 2.2 ± 0.9 | 2.2 ± 0.6 | 1.7 ± 0.7 | 1.9 ± 0.8 | 1.9 ± 0.7 | 2.1 ± 0.8 | 2.4 ± 0.8 | 2.4 ± 0.9 |
| Hours of nightly sleep^1^ | 6.8 ± 1.3 | 7.2 ± 0.7 | 6.9 ± 1.4 | 7.6 ± 0.9 | 7.6 ± 1.2 | 7.5 ± 0.9 | 7.4 ± 0.9 | 7.4 ± 0.9 | 7.1 ± 1.6 |
| Alcohol intake^2^ | 7.6 ± 10.0* | 11.4 ± 17.7 | 8.4 ± 13.8 | 1.8 ± 2.9 | 1.5 ± 2.3 | 1.5 ± 2.5 | 3.7 ± 5.1 | 3.6 ± 4.9 | 3.5 ± 4.7 |
| Fruit/vegetable intake^3^ | 2.5 ± 0.8 | 2.6 ± 0.7 | 2.2 ± 0.6 | 2.7 ± 0.9 | 2.5 ± 0.7 | 2.3 ± 0.6 | 2.2 ± 0.7 | 2.0 ± 0.6 | 2.2 ± 0.7 |
| Whole grain intake^4^ | 0.7 ± 0.4 | 0.7 ± 0.2 | 0.7 ± 0.2 | 0.8 ± 0.3 | 0.8 ± 0.4 | 0.8 ± 0.3 | 0.7 ± 0.3 | 0.6 ± 0.3 | 0.7 ± 0.3 |
| Dairy intake^3^ | 2.1 ± 0.9 | 1.7 ± 0.5 | 1.7 ± 0.6 | 2.2 ± 0.9 | 1.9 ± 0.8 | 1.7 ± 0.6 | 1.8 ± 0.6 | 1.5 ± 0.7 | 1.4 ± 0.4 |
| Red/processed meat intake^4^ | 0.9 ± 0.8 | 1.0 ± 1.1 | 0.9 ± 0.3 | 0.9 ± 0.8 | 0.9 ± 0.8 | 0.7 ± 0.7 | 0.9 ± 0.7 | 0.6 ± 0.7 | 0.6 ± 0.8 |
| Daily sugar intake (g) | 21.5 ± 8.5 | 22.3 ± 14.3 | 20.9 ± 14.8 | 17.7 ± 5.8 | 18.9 ± 6.6 | 16.4 ± 4.4 | 20.3 ± 8.3 | 16.4 ± 7.4 | 17.6 ± 8.1 |
| Daily fiber intake (g) | 16.8 ± 4.2 | 15.2 ± 3.4 | 16.1 ± 3.8 | 17.1 ± 3.5 | 16.3 ± 3.4 | 15.8 ± 2.8 | 15.0 ± 3.3 | 13.8 ± 2.1 | 14.5 ± 3.2 |
| Data displayed as mean ± SD, unless stated otherwise.  Abbreviations: FI, food insecure; FS, food secure; BMI, body mass index; MVPA, Moderate-to-vigorous physical activity.  Superscripts: ^1^Average of combined weekday and weekend nightly sleep hours; ^2^Number of beverages over the last 7 days; ^3^Expressed as daily cup equivalents; ^4^Daily number of servings. *Significant difference compared to FS at Time 1, *P* ≤ 0.05. | | | | | | | | | |

**
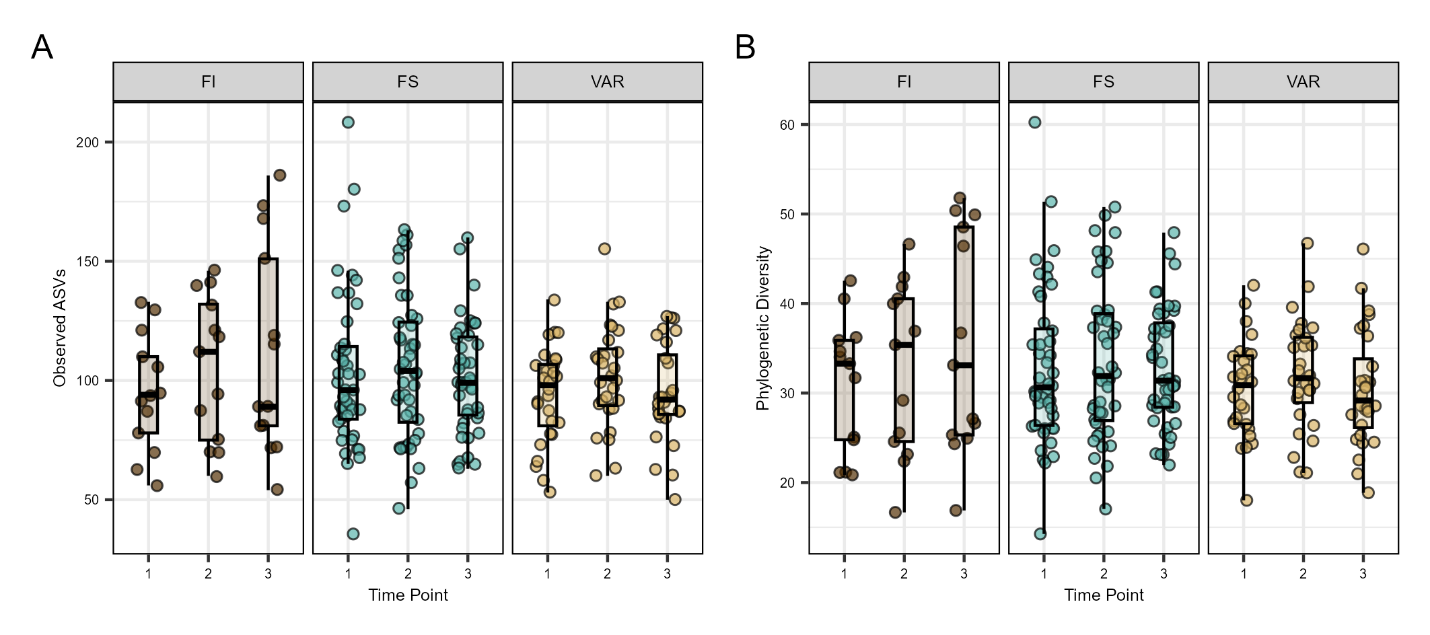
**

**Fig. S1.** Alpha diversity metrics (A) observed amplicon sequence variants and (B) phylogenetic diversity at each of the three sample collection points.


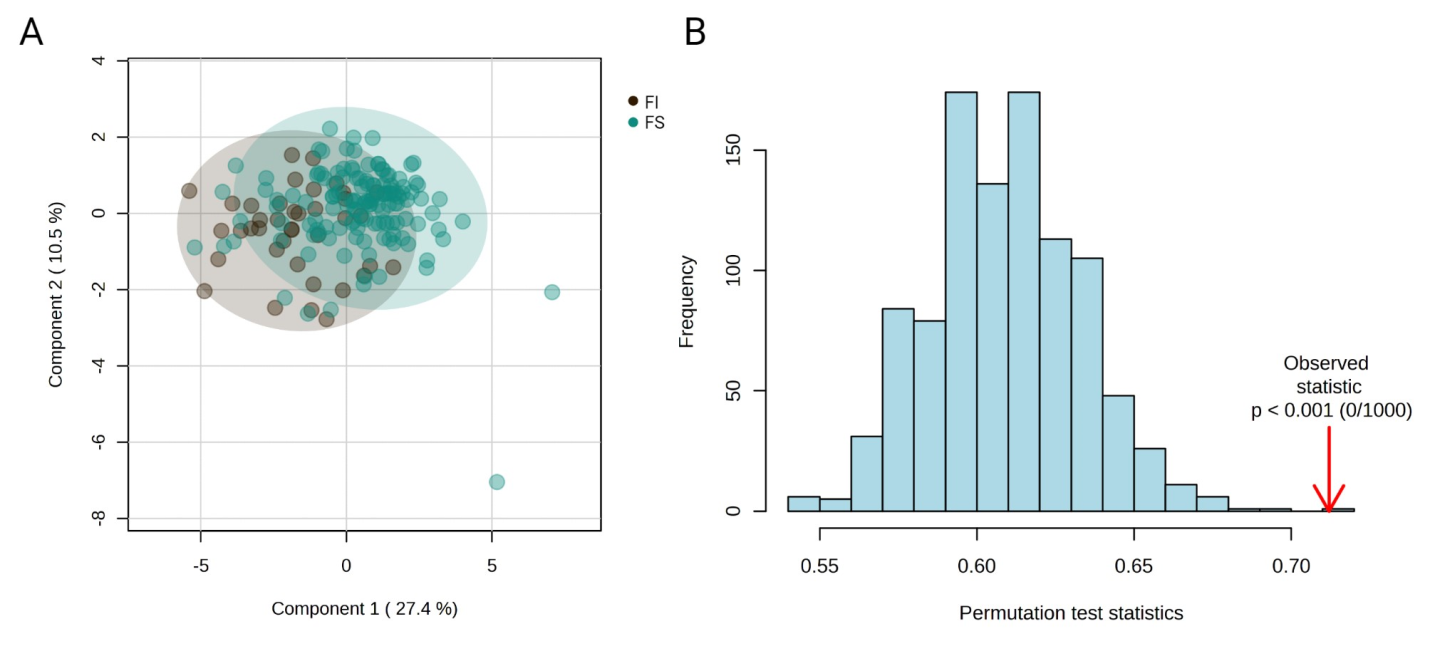


**Fig. S2.** A Partial Least Squares-Discriminant Analysis (PLS-DA) between food insecure (FI) and food secure (FS) individuals using all 26 significant metabolites determined by covariate-controlled general linear modeling. (A) PLS-DA scores plot shows 37.9% of accounted variance between study groups (*R^2^X* = 0.789, *R^2^Y* = 0.308, *R^2^Q* = 0.172; FI, brown; FS, green). (B) Permutation testing by 1000 iterations shows acceptable model fit (observed *P* < 0.001).
